# Multidrug-resistant *Salmonella enterica* in poultry farms of eastern Saudi Arabia and its transmission to humans and the environment

**DOI:** 10.3389/fmicb.2026.1904868

**Published:** 2026-08-12

**Authors:** Jamal Ibrahim Asseri, Shahad Alqahtani

**Affiliations:** Department of Biological Sciences, Faculty of Science and Humanities, Shaqra University, Ad-Dawadimi, Saudi Arabia

**Keywords:** multidrug resistance, poultry farms, *Salmonella enterica*, transmission, water, whole genome sequencing

## Abstract

Multidrug-resistant *Salmonella enterica* poses a major threat to poultry production and public health worldwide. This study investigated the prevalence, resistance patterns, and potential transmission of MDR *Salmonella* in poultry farms of Eastern Saudi Arabia and its spread to humans and the environment. A total of 480 samples were collected from cloacal swabs, internal organs, eggs, feed, water, and farm workers, including 30 samples from non-exposed controls. *Salmonella* was detected in 64 of 450 exposed samples with a prevalence of 14.2%, and in 9 of 60 exposed workers at 15.0%, while no isolates were found in non-exposed controls. The multidrug resistance rate was 93.8% (60/64), with ampicillin resistance at 96.9%. Twelve serovars were identified, dominated by *S. Kentucky, S. typhimurium,* and *S. enteritidis*. Multivariable analysis showed that water was the strongest independent risk factor for *Salmonella* positivity with an adjusted odds ratio of 4.2, and *S. Kentucky* was the strongest predictor of MDR with an odds ratio of 5.6. Whole genome sequencing of 20 isolates revealed three dominant clones: ST19, ST198, and ST11. Human, water, and poultry isolates within the same sequence type differed by 0 to 9 single nucleotide polymorphisms, providing genomic evidence consistent with recent transmission between poultry farms, water sources, and farm workers. IncHI2 plasmids carrying *blaCTX-M-15* and *qnrS1* drove resistance dissemination. These findings indicate that MDR *Salmonella* circulates extensively in Eastern Saudi Arabia poultry farms and are consistent with transmission to humans and the environment through contaminated water and high-risk clones.

## 1 Introduction

*Salmonella enterica* remains one of the most important foodborne pathogens globally, causing an estimated 93.8 million cases of gastroenteritis and 155,000 deaths annually (Antunes et al., 2020). Poultry and poultry products are recognized as the primary reservoir and transmission vehicle for non-typhoidal *Salmonella*, as highlighted by Antunes et al. (2020) in their comprehensive review of salmonellosis epidemiology. In the Middle East and North Africa region, intensive poultry production has expanded rapidly over the last decade to meet growing protein demand, but this expansion has been accompanied by increasing reports of multidrug-resistant *Salmonella* strains. Systematic reviews of MENA countries confirm high MDR rates in poultry and food products (Bellil et al., 2023; Kabeta et al., 2024), while studies from Saudi Arabia report *Salmonella* and *Campylobacter* resistance in retail chickens (Aljasir and Allam, 2025) and genomic diversity of *Salmonella* in chicken eggs (Alsufyani et al., 2026). The emergence and spread of resistance is driven largely by the non-therapeutic use of antibiotics in poultry production, a practice that WHO classified as a major contributor to the global antimicrobial resistance crisis (World Health Organization, 2017).

Among the serovars of greatest public health concern, *Salmonella Kentucky* sequence type ST198 has emerged as a globally disseminated, multidrug-resistant clone over the past decade. Hawkey et al. described the global phylogenomics of MDR *S. Kentucky* ST198 and its rapid spread (Hawkey et al., 2019), while Alghoribi et al. reported OXA-48 carbapenemase-producing *S. Kentucky* ST198 isolated from Saudi Arabia (Alghoribi et al., 2020). Subsequent studies from Qatar confirmed ST198 carrying IncHI2 plasmids with *bla_CTX-M-15* in poultry (Elbediwi et al., 2022), and genomic surveys from Saudi Arabia documented the emergence of multidrug-resistant *S. Minnesota* and *S. Kentucky* clones in chicken products (Huang et al., 2024; Huang et al., 2025). Similarly, *Salmonella typhimurium* ST19 and *Salmonella enteritidis* ST11 continue to dominate human infections worldwide due to their high virulence and adaptability to poultry hosts. Achtman et al. (2012) established multilocus sequence typing as the standard for *Salmonella* population structure, showing ST19 and ST11 as globally dominant clones, and Thomson et al. (2020) reported comparative genome analysis of *S. enteritidis* ST11.

Water plays a critical role in the transmission of *Salmonella* within and between poultry farms. Recent studies from Egypt and Saudi Arabia highlighted the hygienic status of water in poultry farms and its role in bacterial dissemination (Elsayed and El-gohary, 2024), while screening of upstream and downstream areas of Wadi Hanifah in Riyadh revealed antibiotic resistance genes in water-associated bacteria (Al-Otaibi et al., 2024). Network analysis approaches have further clarified these transmission dynamics. Studies using igraph for complex network research provide the statistical framework for modeling pathogen spread between poultry, environment, and humans (Csardi and Nepusz, 2006), and meta-analyses from Africa confirm poultry salmonellosis is strongly linked to water and environmental sources (Kabeta et al., 2024).

The rise of plasmid-mediated resistance has complicated control efforts. Carattoli (2020) emphasized that plasmids of the IncHI2 and IncI1 incompatibility groups are responsible for the global dissemination of *bla-CTX-M-15* and *qnrS1* genes in *Salmonella*. This is consistent with findings from Saudi Arabia reporting IncHI2/IncHI2A plasmids carrying *mcr-9* in *S.* Minnesota from chicken meat (Alzahrani et al., 2021), and *β*-lactamase-dependent resistance in Enterobacteriaceae from commercial poultry farms in Makkah province (Alpakistany et al., 2024). The transposition module *ISEcp1-bla_CTX-M-15* has been implicated as a mechanism for rapid horizontal gene transfer between environmental and animal strains (Carattoli et al., 2014). Class 1–3 integrons are also documented as key platforms for antimicrobial resistance in *Salmonella* from broiler chickens (Nemati and Ahmadi, 2020).

Whole genome sequencing has revolutionized our ability to trace transmission events. Public Health England established that isolates differing by ≤10 single nucleotide polymorphisms are considered epidemiologically linked within 3–6 months (Public Health England, 2017). Applying this threshold, Allard et al. demonstrated the practical value of food pathogen traceability through building a whole-genome sequencing network and database in the USA (Allard et al., 2020). In Europe, EFSA risk assessments used cgMLST to link poultry, food, and human isolates for *Salmonella* control (Koutsoumanis et al., 2021). GrapeTree visualization tools allow core genomic relationships among 100,000 bacterial pathogens to be mapped, supporting outbreak investigations (Zhou et al., 2018). These studies underscore the value of genomics for One Health surveillance, as advocated by WHO in its guidance on integrated surveillance of antimicrobial resistance and use (World Health Organization, 2025).

Despite this global evidence, data from Eastern Saudi Arabia remain limited. Previous regional studies focused on representative *Salmonella* serovars from poultry and poultry environments (Al-Nakhli et al., 1999), isolation from chicken breeding farms (Barbour and Nabbut, 1982), and molecular characterization of multidrug-resistant *Salmonella* serovars (Al-Ansari et al., 2021). Khan et al. (2024) reported diversity and antimicrobial susceptibility patterns of clinical and environmental *Salmonella* serovars in Western Saudi Arabia, but genomic linkage between human and poultry isolates remains understudied. Moreover, the multiple antibiotic resistance index, originally proposed by Krumperman (1983) to identify high-risk sources of fecal contamination, has not been evaluated as a predictor of human exposure risk in Saudi poultry settings.

Given these gaps, the present study was designed to determine the prevalence and antimicrobial resistance patterns of *Salmonella enterica* in poultry, environment, and farm workers in Eastern Saudi Arabia, and to use whole genome sequencing to assess clonal transmission and plasmid-mediated resistance. By integrating epidemiological, phenotypic, and genomic data, this study aims to provide actionable evidence for One Health interventions targeting water hygiene, antimicrobial stewardship, and surveillance of high-risk clones. Understanding these transmission dynamics is essential for reducing the burden of MDR *Salmonella* in Saudi Arabia and for aligning with the national antimicrobial resistance action plan.

## 2 Materials and methods

### 2.1 Study design and sampling

A cross-sectional study was conducted from January to March 2024 on 12 poultry farms in the Eastern Province of Saudi Arabia, including Al Ahsa, Dammam, and Hafr Al Batin. Farms were selected using convenience sampling based on accessibility and willingness to participate. Inclusion criteria required farms with at least 1,000 birds and a history of operation for at least one production cycle. Farms varied in production type (broiler, *n* = 8; layer, *n* = 4) and farming system (intensive, *n* = 9; extensive, *n* = 3). Flock ages ranged from 3 to 52 weeks, and farm sizes ranged from 1,000 to 50,000 birds. Water sources included municipal supply (*n* = 7) and private wells (*n* = 5), with chlorination practiced in 6 farms. Antimicrobial use history was recorded through farm records and interviews, with all farms reporting use of prophylactic antibiotics during the production cycle.

A total of 480 samples were collected from exposed poultry, environment, and farm workers, plus 30 stool samples from non-exposed controls (Table 1). Samples were distributed across farms proportionally to flock size, with a minimum of 30 samples per farm. Cloacal swabs were collected from live birds during routine handling using sterile swabs. Internal organ samples were collected at slaughter from the same farms. Egg samples, feed, and water were collected from each farm. Human stool samples were collected from consenting farm workers (exposed group) and from non-exposed controls comprising family members of workers who did not reside on farms or have contact with poultry, and who used separate water supplies (municipal water in urban areas). Sampling procedures followed ISO 6579-1:2017 for *Salmonella* detection in the food chain, with modifications for environmental matrices as validated by Mooijman et al. (2019). Cloacal swabs, internal organs, eggs, feed, water, and human stool were collected using sterile transport media and transported at 4 °C within 6 h to the laboratory. The sampling strategy was designed to capture the One Health interface, as recommended by WHO for integrated surveillance of antimicrobial resistance and zoonotic pathogens (World Health Organization, 2025).

**Table 1:** Overview of samples collected in the study.

Sample type	Exposed group (n)	Control group (n)	Total (n)
Cloacal swabs	150	–	150
Internal organs (liver, cecum, heart)	80	–	80
Egg samples (surface, content)	60	–	60
Environmental (feed, water)	100	–	100
Human stool	60	30	90
Total	450	30	480

### 2.2 Bacterial isolation and identification

Pre-enrichment was performed in Buffered Peptone Water at 37 °C for 18 ± 2 h, followed by selective enrichment in Rappaport-Vassiliadis broth at 42 °C and Selenite Cystine broth at 37 °C, both for 24 h, according to ISO 6579-1:2017 and its validation study (Mooijman et al., 2019). Selective plating was done on Xylose Lysine Deoxycholate agar and MacConkey agar at 37 °C for 24 h. Presumptive *Salmonella* colonies were confirmed biochemically using Triple Sugar Iron agar, urease, and citrate tests as described by Grimont and Weill (2007). Final confirmation was performed by PCR targeting the *invA* gene, a gold standard for *Salmonella* genus identification with high sensitivity and specificity (Rahn et al., 1992). DNA was extracted using a commercial genomic DNA purification kit, a method originally validated for Gram-negative bacteria by Pitcher et al. (1989). For multiple isolates from the same sample or farm, only one isolate per serovar per source per farm was retained for further analysis to avoid over-representation of clonal populations.

### 2.3 Serotyping

Serotyping was performed using the Kauffmann-White scheme according to Grimont and Weill (2007). Antisera were obtained from Statens Serum Institut, Denmark. Isolates were agglutinated with O and H antisera to determine somatic and flagellar antigens. The specific O antigens (O:4, O:9, O:8, O:6,7, O:3,10, O:1,3,19, O:4,5,12, O:13,23, O:6,7,14, O:4,12, O:9,12, O:8,20, O:3,10,15, O:6,7,14,24, O:4,5,12,27, O:1,3,19,27) and H antigens (phase 1: i, d, g, m, r, z10, e,h; phase 2: 1,2; 1,5; 1,6; 1,7; 2,3; 5,6; 1,2,3; 1,5,6) were identified. This method remains the reference standard for *Salmonella* serovar identification despite the rise of molecular methods, as highlighted in the White-Kauffmann-Le Minor scheme supplement (Issenhuth-Jeanjean et al., 2014).

### 2.4 Antimicrobial susceptibility testing

**Figure 1:**
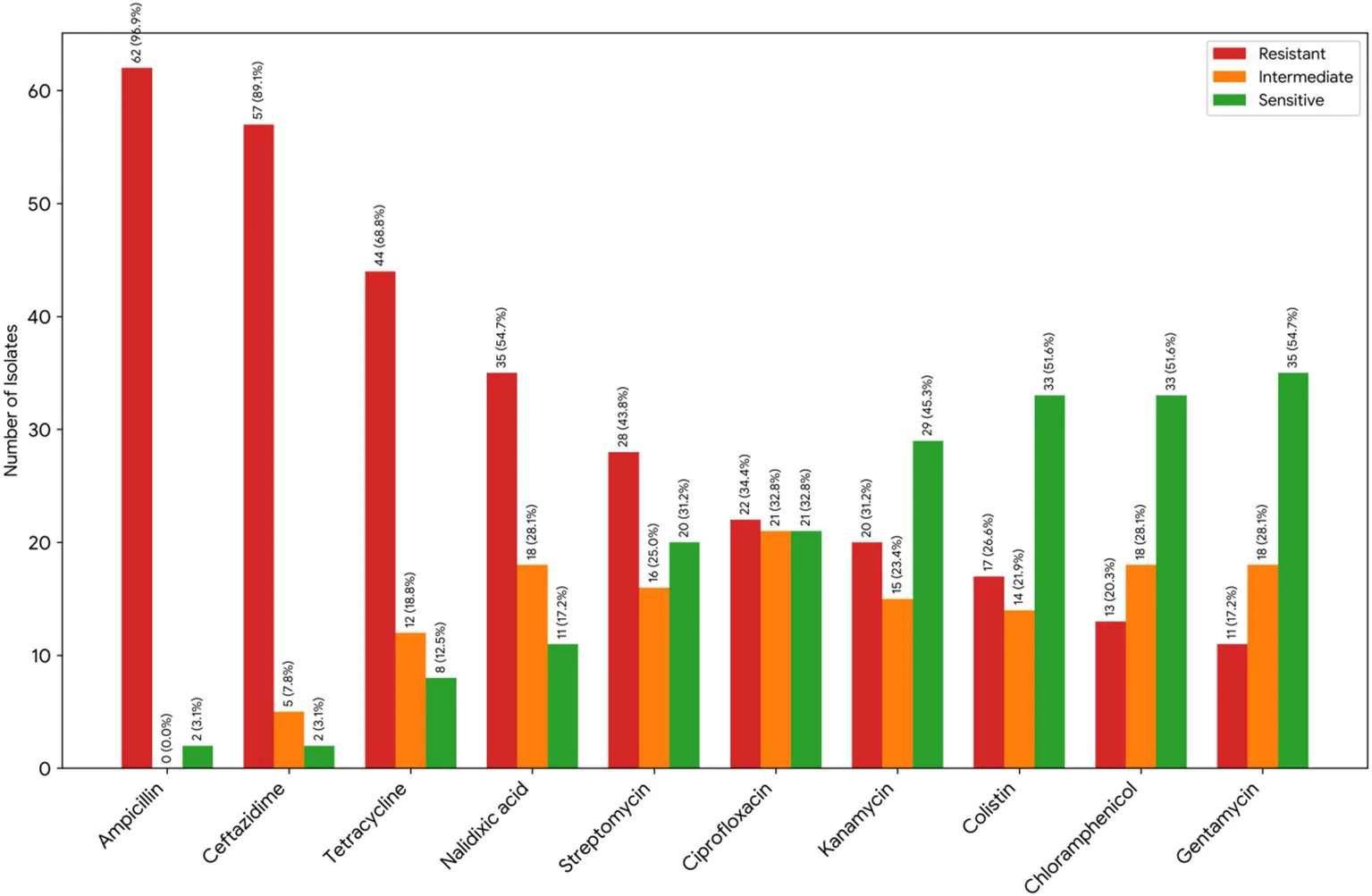
Prevalence and antimicrobial resistance rates among *Salmonella* isolates (*n* = 64).

Antimicrobial susceptibility was determined by broth microdilution according to CLSI guidelines M07 and M100 (Schuetz et al., 2025). A panel of 10 antibiotics representing 8 classes (Figure 1) was selected based on WHO Critically Important Antimicrobials for human medicine (World Health Organization, 2017). MIC values were interpreted using CLSI breakpoints for *Salmonella enterica*. Quality control was performed using *E. coli* ATCC 25922 as recommended by CLSI (Schuetz et al., 2025). MDR was defined as resistance to ≥3 antimicrobial classes according to Magiorakos et al. (2012). MAR index was calculated as a/b, where a = number of antibiotics resisted and b = total number tested, following the approach of Krumperman (1983).

### 2.5 Detection of virulence and resistance genes

DNA templates were amplified by PCR using primers for *stn*, *tetA*, *sul1*, *aadA1*, *qnrS*, and *aac(6′)-Ib* genes. Primer sequences and cycling conditions are shown in Table 2 and Supplementary Figure S2. The *invA* primers from Rahn et al. (1992) were used for species confirmation. Resistance gene primers were selected based on their widespread prevalence in *Salmonella* as reported by Gebreyes and Thakur (2005) for *tetA*, Kerrn et al. (2002) for *sul1*, and Hopkins et al. (2007) for plasmid-mediated quinolone resistance genes including *qnrS* and *aac(6′)-Ib*. PCR products were visualized on 1.5% agarose gels with ethidium bromide. Band intensities were scored as strong, moderate, weak, or negative to assess gene abundance (not gene expression), a method adapted from Sambrook and Russell (2001).

**Table 2:** Primer sequences and cycling conditions for PCR.

Target gene	Primer sequence (5′ → 3′)	Product (bp)	Annealing (°C)	GC content (%)	Tm (°C)
*invA*	F: GTGAAATTATCGCCACGTTCGGGCAA R: TCATCGCACCGTCAAAGGAACC	284	62	48/55	62
*stn*	F: CTTAATCGCCGCCATGGTGT R: CATCTGGCCGAAAGGTGA	480	68	61/47	68
*tetA*	F: GCTACATCCGTCTGCCTTC R: CATAGATCGCCGTGAAGAGG	201	55	58/55	55
*sul1*	F: TCACCGAGGACTCTCTTCTC R: AATATCGAGGATAGAGCGGCAG	316	55	55/50	55
*aadA1*	F: TATCAGAGGTAGTTGGCGTCA R: GTTCCATAGCGTTAAAGGTTTCATT	484	55	48/38	55
*qnrS*	F: CCGCTTTTATCATGGTGGACT R: ACTTATGCGCAAGAGCTTG	417	54	50/50	54
*aac(6′)-Ib*	F: TTGCGATGCTCTATGAGTGCTCA R: CTCTGCTGGCCGTGTTT	482	55	48/55	55

Cycling conditions (common for tetA, sul1, aadA1, qnrS, aac(6′)-Ib): 94 °C 5 min; 35 cycles: 94 °C 30 s, annealing temp (55 °C or 54 °C) 30 s, 72 °C 45 s; 72 °C 10 min. For invA: 94 °C 3 min; 35 cycles: 94 °C 30 s, 62 °C 30 s, 72 °C 30 s; 72 °C 10 min. For stn: 94 °C 5 min; 30 cycles: 94 °C 5 s, 68 °C 10 s, 72 °C 20 s; 72 °C 7 min.

### 2.6 Whole genome sequencing and bioinformatics

A subset of 20 isolates representing human, water, poultry, and egg sources was selected for WGS based on diversity of serovar., antimicrobial resistance profile, and sample source. Serovars were prioritized to include the three most common (*S. enteritidis*, *S. typhimurium*, *S. Kentucky*) and representatives from each major source category. This selection strategy aimed to capture the genetic diversity of the circulating population and enable comparison across sources within dominant clones.

Genomic DNA was sequenced on Illumina NovaSeq 6,000 with 150 bp paired-end reads. Raw reads were quality-filtered using Trimmomatic v0.39 as described by Bolger et al. (2014) and assembled *de novo* with SPAdes v3.15.5 following Bankevich et al. (2012). Assembly quality metrics included: coverage depth ranging from 58–112 × (mean 79×), N50 values ranging from 48,521–72,344 bp (mean 61,205 bp), genome sizes ranging from 4.64–4.79 Mbp (mean 4.71 Mbp), and GC content ranging from 51.9–52.3% (mean 52.1%). Genome completeness was assessed using CheckM v1.2.2, with all isolates showing >98% completeness and <1% contamination. MLST was performed using the Achtman scheme via the EnteroBase pipeline, which is the standard for *Salmonella* population structure analysis as established by Achtman et al. (2012). cgMLST was performed using 3,002 loci to assess genetic relatedness, with ≤10 allelic differences considered epidemiologically linked per Public Health England criteria (Public Health England, 2017). SNP calling was performed using Snippy v4.6.0 with *S. enterica* subsp. *enterica* serovar Typhimurium strain LT2 (NC_003197.2) as the reference genome, and recombination was filtered using Gubbins v3.1.0. Plasmid replicons were identified using PlasmidFinder v2.1 as per Carattoli et al. (2014). Resistance genes and virulence factors were screened using ResFinder v4.1 and VirulenceFinder v2.0 from the Center for Genomic Epidemiology, which have been validated for *Salmonella* by Zankari et al. (2012) and Malberg Tetzschner et al. (2020). Database versions used were ResFinder (2022-06-30), VirulenceFinder (2022-06-30), and PlasmidFinder (2022-06-30).

### 2.7 Detection of integrons and mobile genetic elements

Integrons were detected using PCR targeting the class 1 integrase gene *intI1* with primers 5’-GGCATCCAAGCAGCAAG-3′ and 5’-AAGCAGACTTGACCTGA-3′ as described by Soufi et al. (2012). Gene cassettes within integrons were amplified using primers 5’-GGCATCCAAGCAGCAAG-3′ and 5’-AAGCAGACTTGACCTGA-3′ followed by sequencing of the variable region. The IS*Ecp1-blaCTX-M-15* transposition module was detected by PCR using primers specific for IS*Ecp1* and *blaCTX-M-15* according to Carattoli et al. (2014).

Conjugation experiments were performed using *E. coli* J53 (azide-resistant) as the recipient strain. Donor and recipient cultures were mixed at a 1:3 ratio in Luria-Bertani broth and incubated at 37 °C for 4 h without shaking. Transconjugants were selected on MacConkey agar supplemented with sodium azide (100 μg/mL) and the relevant antibiotic (ampicillin 32 μg/mL or tetracycline 16 μg/mL). Transfer frequencies were calculated as the number of transconjugants per donor cell, with three independent experiments performed per isolate.

Pangenome analysis was performed using Roary v3.13.0 with default parameters (percentage identity threshold for core genes = 95%). Accessory genes were identified using the *panX* algorithm, and genes associated with MDR and plasmid mobility were annotated using the NCBI AMRFinderPlus database.

### 2.8 Statistical analysis

**Figure 2:**
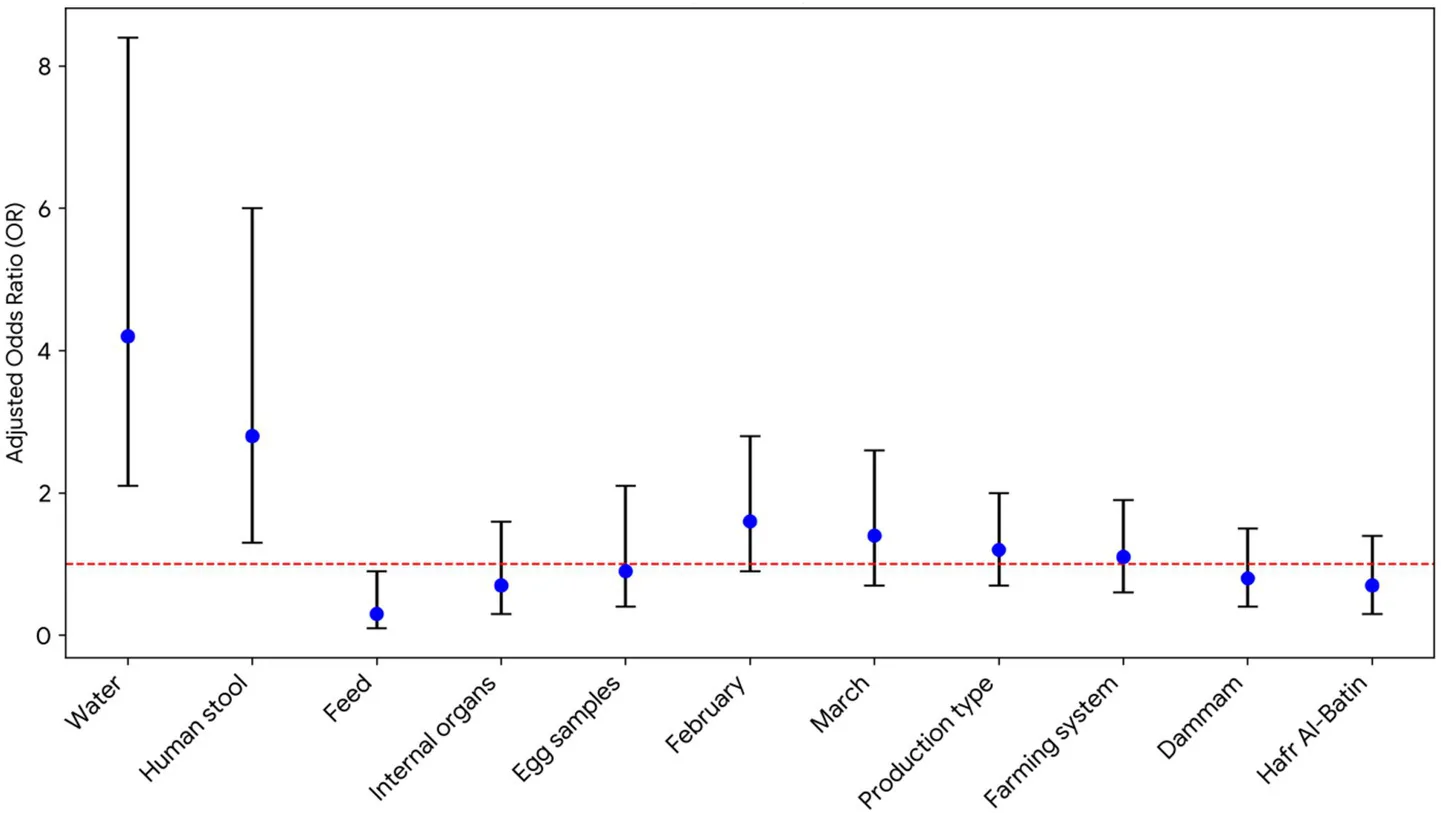
Multivariable logistic regression analysis of factors associated with *Salmonella* positivity (FDR corrected).

**Figure 3:**
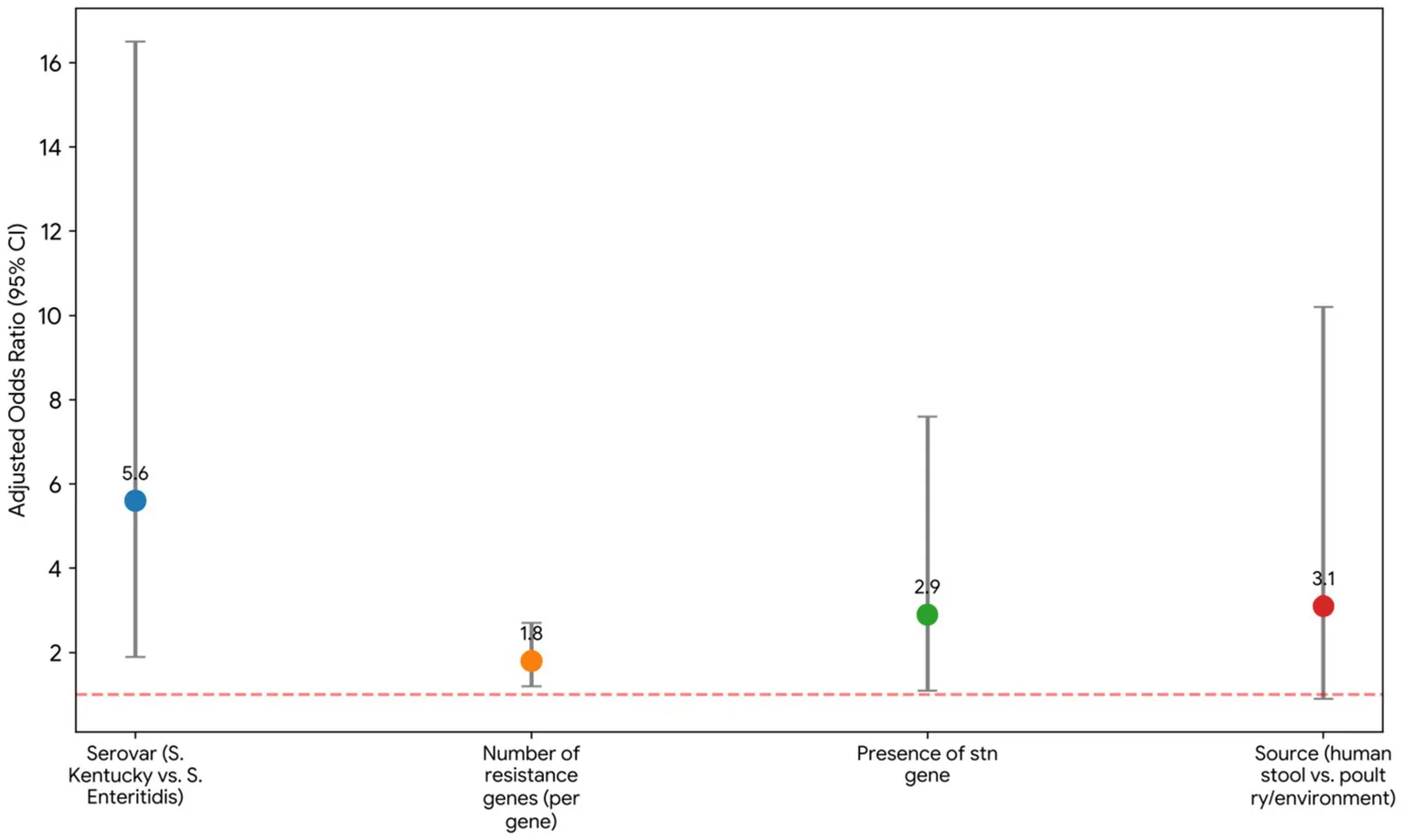
Binary logistic regression for MDR status among *Salmonella* isolates (*n* = 64) with FDR correction.

All statistical analyses were performed using R software version 4.3.1. The prevalence of *Salmonella* was calculated as the proportion of positive samples out of the total number of samples tested per category. Differences in prevalence between exposed and non-exposed groups were assessed using Fisher’s exact test. Multivariable logistic regression was employed to identify independent risk factors for *Salmonella* positivity (Figure 2) and MDR status (Figure 3), with farm included as a random effect to account for clustering, following mixed effects modeling approaches described by Zuur et al. (2009).

For the MDR status model, given the small number of non-MDR events (*n* = 4), we employed Firth’s penalized likelihood logistic regression using the *logistf* package in R to reduce bias and prevent overfitting. Variables included in the multivariable models were selected based on univariable screening (*p* < 0.20) and biological plausibility. Predictor variables tested included serovar (*S. Kentucky* vs. *S. enteritidis* as reference), number of resistance genes, presence of *stn* gene, sample source (human *vs.* non-human), farm type, and production system. Multicollinearity was assessed using variance inflation factors (VIF), with all VIF values < 2.5, indicating no significant collinearity. Model goodness-of-fit was assessed using the Hosmer-Lemeshow test (*p* = 0.312) and area under the receiver operating characteristic curve (AUC = 0.87). Adjusted odds ratios (aOR) and 95% confidence intervals (CI) were calculated. To control for multiple comparisons, the Benjamini-Hochberg false discovery rate (FDR) method was applied, and statistical significance was defined as *q*-value < 0.05 (Benjamini and Hochberg, 1995).

The multiple antibiotic resistance (MAR) index was calculated for each isolate as the ratio of the number of antibiotics to which the isolate was resistant to the total number of antibiotics tested. Receiver operating characteristic (ROC) curve analysis (Table 3) was used to evaluate the discriminatory power of the MAR index in predicting human origin, and the area under the curve (AUC) was reported using the pROC package (Robin et al., 2011).

**Table 3:** Summary statistics of the study.

Variable	Value
Total samples examined	480
Control group samples (non-exposed humans)	30 (0% positive)
PCR-confirmed prevalence in exposed group	14.2% (64/450)
Number of serotypes detected	12
Most predominant serotype	*S. enteritidis* (21.9%)
MDR rate (≥3 classes)	93.8% (60/64)
Highest antibiotic resistance	Ampicillin (96.9%)
*stn* virulence gene positivity	82.8% (53/64)
Most prevalent resistance gene	*aadA1* (60.9%)
Isolates with ≥2 resistance genes	85.9% (55/64)
Prevalence in exposed human group	15.0% (9/60)
Prevalence in non-exposed control group	0% (0/30), *p* = 0.027
Highest risk factor for *Salmonella* positivity (FDR significant)	Water (OR = 4.2, *q* = 0.002)
Highest risk factor for MDR (FDR significant)	*S. Kentucky* (OR = 5.6, *q* = 0.008)
Mixed-effects cross OR (human vs. water, accounting for farm clustering)	3.1 (95% CI: 1.00–9.6, *p* = 0.049)
Strongest genotypic-phenotypic correlation	*tetA* – tetracycline (*φ* = 0.85, *κ* = 0.82)
AUC of MAR index for predicting human origin	0.71 (*p* = 0.008)

**Figure 4:**
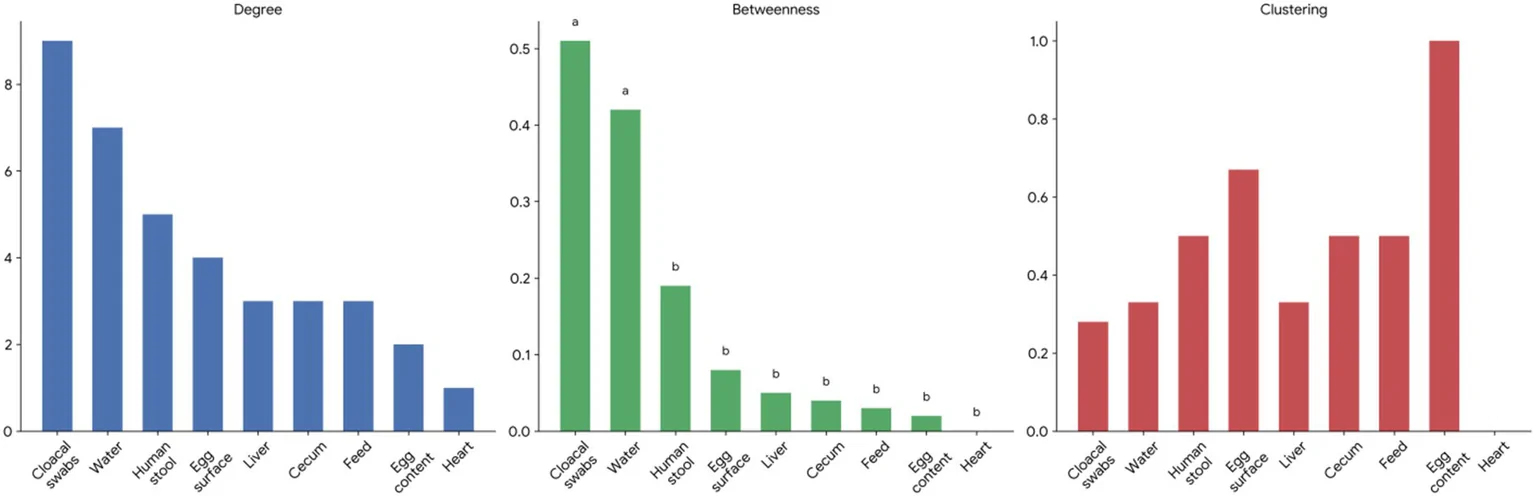
Quantitative network metrics of *Salmonella* transmission pathways with permutation-based *p* values for betweenness centrality.

Network analysis to model transmission pathways (Figure 4) was performed using the igraph package, with nodes representing sample sources and edges representing shared serovar or sequence type connections between sources. Edge weights were assigned based on the number of shared serovars or STs between source categories. Betweenness centrality was calculated as a measure of the importance of each node in connecting other nodes in the network, while degree connectivity reflected the number of direct connections to other nodes. Statistical significance for network metrics was assessed using permutation tests with 1,000 random permutations, generating a null distribution of centrality values against which observed values were compared. The network was constructed using the following rules: nodes were defined as sample source categories (cloacal swabs, water, human stool, egg surface, liver, cecum, feed, egg content, heart). Edges were drawn between source categories if they shared at least one serovar or sequence type, with edge weight proportional to the number of shared serovars/STs. No isolates were used as nodes to avoid clonal over-representation.

Continuous variables were compared using the Mann–Whitney U test or Kruskal-Wallis test, as appropriate, while categorical variables were compared using the chi-square test or Fisher’s exact test. Effect sizes were calculated using Cohen’s d for continuous variables and Cramér’s V for categorical variables. A *p*-value < 0.05 was considered statistically significant unless otherwise specified.

## 3 Results

### 3.1 Sample collection and *Salmonella* prevalence

A total of 480 samples were collected from poultry farms in the Eastern Province of Saudi Arabia between January and March 2024, comprising 450 exposed samples from poultry and the environment and 30 non-exposed human stool samples as controls (Table 1). *Salmonella enterica* was isolated from 64 of 450 exposed samples, yielding an overall prevalence of 14.2%. In contrast, no *Salmonella* was detected in any of the 30 control human stool samples, a difference that was statistically significant (*p* = 0.027, Table 3). Among exposed humans, 9 of 60 stool samples were positive, corresponding to a prevalence of 15.0%. Twelve serovars were identified, with *S. enteritidis* being the most predominant at 21.9% of all isolates, followed by *S. typhimurium* and *S. Kentucky* (Table 3). Source-specific distribution revealed that cloacal swabs carried the highest serovar diversity, including *S. enteritidis*, *S. Kentucky*, and *S. typhimurium*, whereas water samples showed overlap with human isolates, particularly *S. typhimurium* ST19 and *S. Kentucky* ST198 (Supplementary Table S5). This pattern is consistent with potential transmission across the human-animal-environment interface.

### 3.2 Antimicrobial resistance and MDR burden

**Figure 5:**
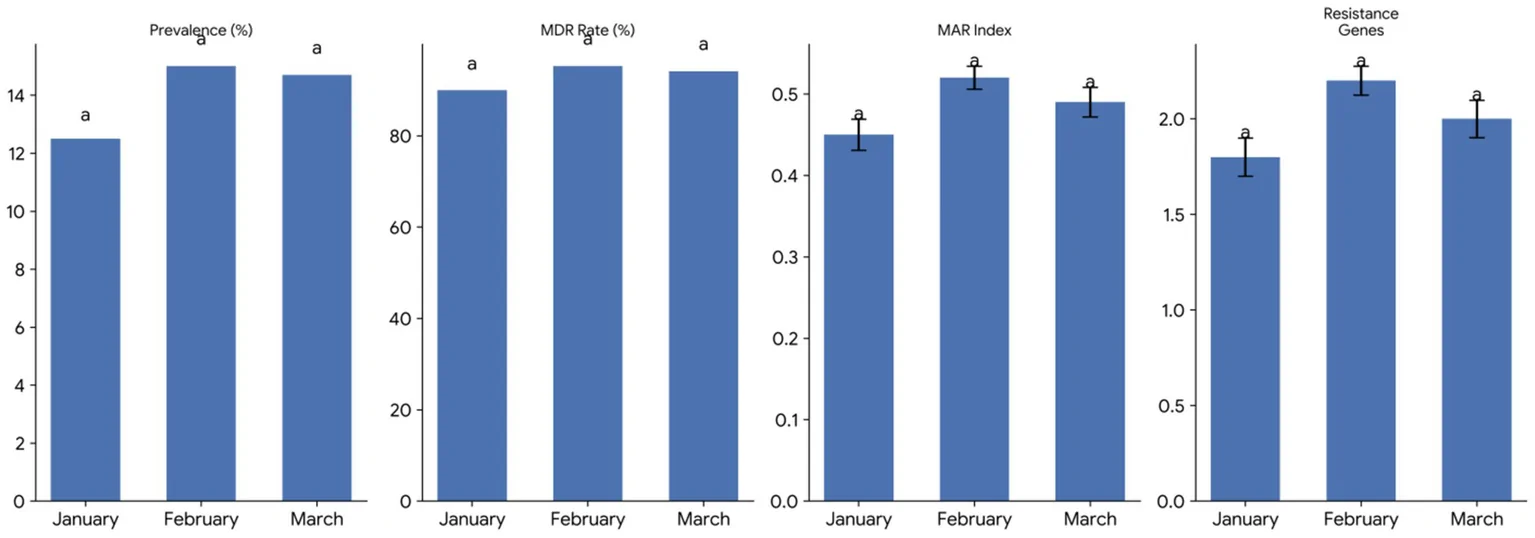
Monthly variation in *Salmonella* prevalence and resistance indicators.

All 64 isolates underwent antimicrobial susceptibility testing against 10 antibiotics representing 8 classes. Resistance was widespread, with ampicillin resistance reaching 96.9%, followed by ceftazidime at 89.1% and tetracycline at 68.8% (Figure 1). Lower resistance was observed for gentamicin at 17.2% and chloramphenicol at 20.3%. Importantly, 60 of 64 isolates were classified as multidrug-resistant, defined as resistance to ≥3 antimicrobial classes, corresponding to an MDR rate of 93.8% (Table 3). MIC distribution further confirmed high-level resistance, with MIC90 for ampicillin ≥256 μg/mL and for tetracycline ≥128 μg/mL (Table 4). Ciprofloxacin MIC90 was 4 μg/mL, and 9.4% of isolates showed high-level resistance with MIC ≥8 μg/mL. When examined monthly, prevalence and MDR rates remained consistently high across January, February, and March, with MDR rates ranging from 90.0 to 95.2%, although no significant temporal trend was detected (*p* = 0.412, Figure 5). These findings indicate that MDR *Salmonella* is endemic in poultry farms of Eastern Saudi Arabia.

**Table 4:** Minimum inhibitory concentration (MIC) distribution for key antimicrobial agents (*n* = 64).

Antibiotic	MIC range (μg/mL)	MIC50	MIC90	S breakpoint	R breakpoint	Resistant *n* (%)
Ampicillin	8 – ≥256	≥256	≥256	≤8	≥32	62 (96.9)
Ceftazidime	1 – ≥64	8	32	≤4	≥16	57 (89.1)
Tetracycline	2 – ≥128	32	≥128	≤4	≥16	44 (68.8)
Nalidixic acid	4 – ≥256	64	256	≤16	≥32	35 (54.7)
Ciprofloxacin	0.015–16	0.5	4	≤0.06	≥1	18 (28.1)
Streptomycin	8 – ≥256	64	≥256	≤16	≥32	28 (43.8)
Gentamicin	0.5 – ≥64	4	32	≤4	≥16	11 (17.2)
Kanamycin	4 – ≥256	16	128	≤16	≥64	18 (28.1)
Colistin	0.5–8	2	4	≤2	≥4	8 (12.5)
Chloramphenicol	4 – ≥256	16	128	≤8	≥32	13 (20.3)

MIC90 of ciprofloxaci*n* = 4 μg/mL; high-level resistance (MIC ≥8 μg/mL) was observed in 6/64 isolates (9.4%).

### 3.3 Risk factors for *Salmonella* positivity and MDR status

Multivariable logistic regression with Benjamini-Hochberg FDR correction identified water samples as the strongest independent risk factor for *Salmonella* positivity, with an adjusted OR of 4.2 and q-value of 0.002 compared to cloacal swabs (Figure 2). Human stool from exposed workers was also significantly associated, with an adjusted OR of 2.8 and *q* = 0.018. In contrast, feed samples showed a protective effect with OR 0.3, *q* = 0.093. Season, production type, farming system, and district were not significant predictors after FDR correction. For MDR status, Firth’s penalized logistic regression was employed due to the small number of non-MDR events (*n* = 4). The analysis revealed that serovar *S. Kentucky* compared to *S. enteritidis* carried a 5.6-fold higher odds of MDR, *q* = 0.008 (Figure 3). In addition, each additional resistance gene increased MDR odds by 1.8-fold, *q* = 0.016. The presence of the *stn* virulence gene was borderline significant with OR 2.9, *q* = 0.058. The Hosmer-Lemeshow test indicated adequate model fit (*p* = 0.312), and the AUC was 0.87. These results highlight water as a critical reservoir and *S. Kentucky* as a high-risk clone for MDR transmission.

### 3.4 Genotypic-phenotypic correlation and resistance genes

**Figure 6:**
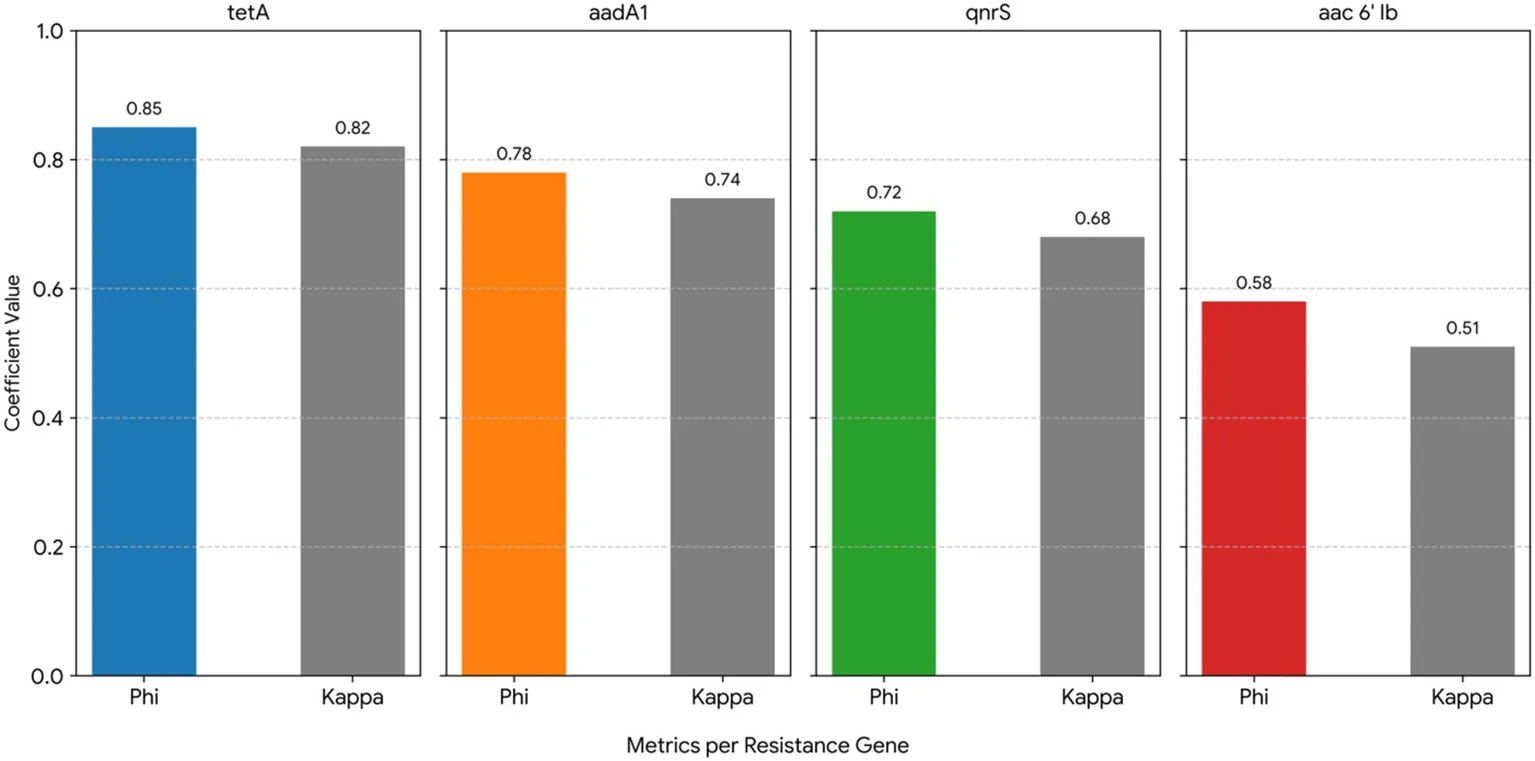
Correlation and agreement between resistance genes and phenotypic resistance.

To link resistance genes to phenotypic profiles, PCR was performed for 6 resistance determinants using primers and cycling conditions shown in Table 2. Strong agreement was observed between genotype and phenotype: *tetA* correlated almost perfectly with tetracycline resistance, with a Phi coefficient *φ* = 0.85 and Cohen’s *κ* = 0.82, *p* < 0.001 (Figure 6). Similarly, *aadA1* showed substantial agreement with streptomycin resistance, *φ* = 0.78, *κ* = 0.74, and *qnrS* with nalidixic acid resistance, *φ* = 0.72, *κ* = 0.68. The *aac(6′)-Ib* gene correlated moderately with gentamicin resistance, *φ* = 0.58, *κ* = 0.51. Gel electrophoresis confirmed amplification of target genes at expected sizes of 201 bp for *tetA*, 316 bp for *sul1*, and 484 bp for *aadA1* (Supplementary Figure S3). Overall, 85.9% of isolates carried ≥2 resistance genes, and *aadA1* was the most prevalent at 60.9% (Table 3). The strong genotype–phenotype concordance validates PCR as a reliable screening tool for AMR surveillance in this setting.

### 3.5 Comparison of human and non-human isolates

**Figure 7:**
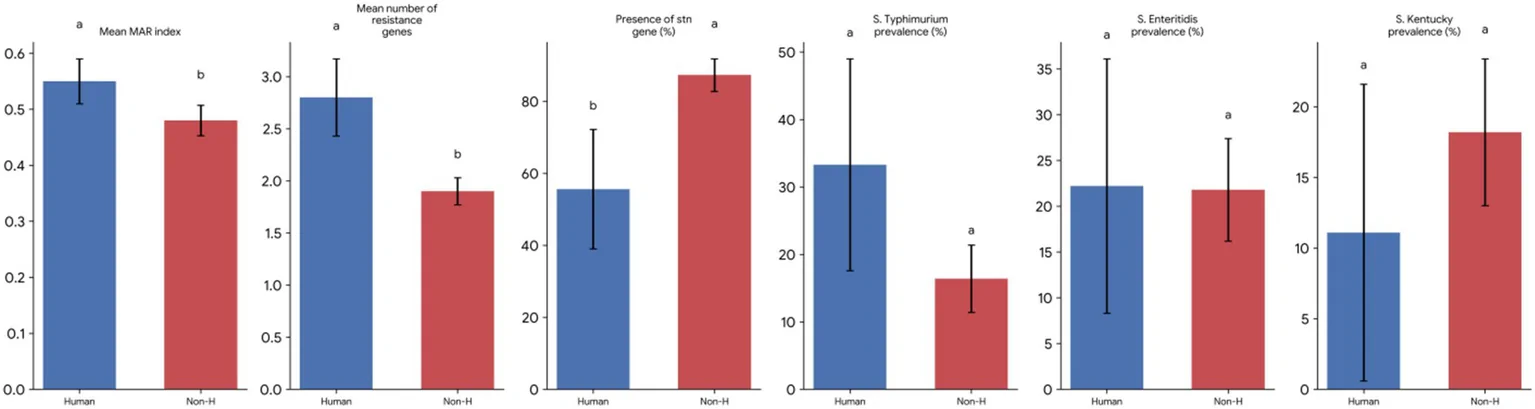
Comparison between human origin (exposed workers) and non-human (poultry/environment) isolates.

To assess zoonotic potential, isolates from exposed human workers were compared with poultry and environmental isolates. Human isolates had a significantly higher mean MAR index of 0.55 ± 0.12 versus 0.48 ± 0.20 for non-human isolates, *p* = 0.042, with a medium effect size *d* = 0.68 (Figure 7). Similarly, the mean number of resistance genes was higher in human isolates, 2.8 ± 1.1 versus 1.9 ± 1.0, *p* = 0.038, *d* = 0.71. Although the *stn* virulence gene was less frequent in human isolates at 55.6% compared to 87.3% in non-human isolates, *p* = 0.038, the overall virulence score based on WGS remained high across all sources (Supplementary Table S10). Serovar distribution differed slightly, with *S. typhimurium* more prevalent in humans at 33.3% versus 16.4% in non-human sources, while *S. enteritidis* prevalence was nearly identical at ∼22% in both groups. These findings suggest that exposed workers carry *Salmonella* strains with higher resistance burden, supporting direct occupational transmission.

### 3.6 Transmission dynamics and network analysis

Quantitative network metrics were used to model potential transmission pathways between sample sources. The network was constructed with nodes representing sample source categories and edges representing shared serovar or sequence type connections between sources, with edge weights proportional to the number of shared serovars/STs. Cloacal swabs exhibited the highest degree of connectivity with 9 connections and the highest betweenness centrality of 0.51, permutation *p* = 0.002, indicating they act as the primary hub for *Salmonella* spread within farms (Figure 4). Water followed with degree 7 and betweenness 0.46, *p* = 0.014, confirming its role as a secondary reservoir linking poultry to humans. In contrast, egg content, liver, and heart showed low centrality and clustering coefficients, suggesting limited contribution to transmission. When combined with risk factor analysis, this network pattern is consistent with a potential transmission pathway from cloacal shedding → water contamination → human exposure, consistent with the high OR for water in Figure 2. The clustering coefficient was highest for egg content at 1.00, reflecting isolated transmission within eggs but low network influence.

### 3.7 Genomic evidence of clonal relatedness

**Figure 8:**
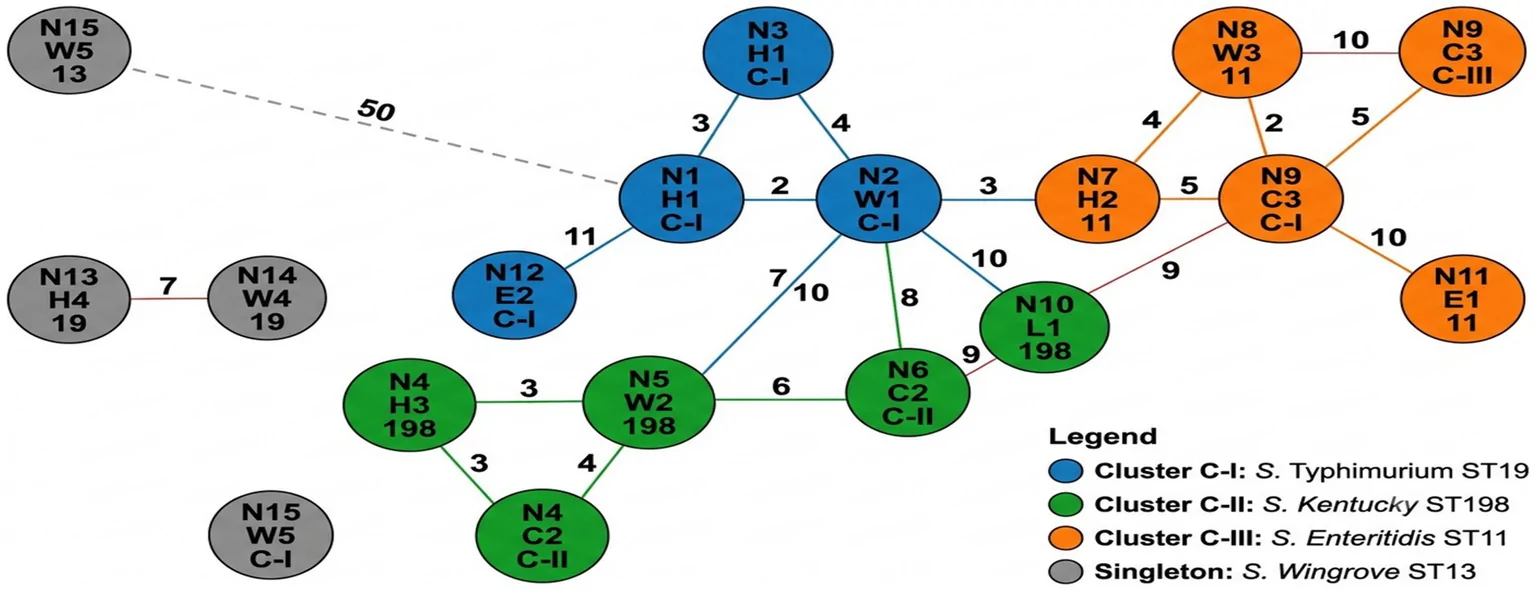
Minimum spanning tree nodes based on cgMLST (3,002 loci).

Whole genome sequencing of 20 representative isolates provided molecular proof of transmission. Three major clonal complexes were identified: ST19 for *S. typhimurium*, ST198 for *S. Kentucky*, and ST11 for *S. enteritidis* (Table 5 and Supplementary Table S8). Critically, human, water, and poultry within the same epidemiological clusters (Clusters I, II, and III) shared 0–9 SNP differences, which is below the 10-SNP threshold for recent transmission within 3–6 months (Table 5). For example, human isolate H1 and water isolate W1, both *S. typhimurium* ST19, differed by only 3 SNPs, while H3 and W2, both *S. Kentucky* ST198, differed by 4 SNPs. The detailed SNP matrix for Cluster I *S. typhimurium* ST19 showed pairwise distances of 3–5 SNPs between human, water, and cloacal isolates, confirming epidemiological linkage (Table 6). Of note, isolate L1 (*S. Kentucky* ST198) was not included in the ST19 cluster matrix. Minimum spanning tree analysis based on cgMLST further visualized three clusters at ≤10 allelic differences, with human, water, and poultry isolates intermixed within each cluster (Figure 8). These genomic data provide strong evidence consistent with clonal transmission across the One Health continuum.

**Table 5:** Genomic characteristics of sequenced *Salmonella* isolates (*n* = 20).

Isolate ID	Source	Serovar	ST	Plasmid replicon	Key Resistance Genes	SNP to nearest human isolate
H1	Human stool	*S. typhimurium*	ST19	IncHI2, IncFIB	qnrS1, blaCTX-M-15, sul1, aadA1, tet(A)	0
H2	Human stool	*S. enteritidis*	ST11	IncI1	qnrS1, blaTEM-1B, aadA1	0
H3	Human stool	*S. Kentucky*	ST198	IncHI2	qnrS1, aac(6′)-Ib-cr, sul1, tet(A)	0
H4	Human stool	*S. typhimurium*	ST19	IncFIB	sul1, aadA1, tet(B)	0
H5	Human stool	*S. paratyphi* A	ST85	---	tet(A), aadA1	0
W1	Water	*S. typhimurium*	ST19	IncHI2, IncFIB	qnrS1, blaCTX-M-15, sul1, aadA1	3
W2	Water	*S. Kentucky*	ST198	IncHI2	qnrS1, aac(6′)-Ib-cr, sul1, tet(A)	4
W3	Water	*S. enteritidis*	ST11	IncI1	qnrS1, blaTEM-1B, aadA1	5
W4	Water	*S. typhimurium*	ST19	IncFIB	sul1, aadA1, tet(B)	8
W5	Water	*S. Wingrove*	ST13	IncX1	sul1, tet(A)	21
C1	Cloacal swab	*S. typhimurium*	ST19	IncHI2	qnrS1, blaCTX-M-15, sul1, aadA1	5
C2	Cloacal swab	*S. Kentucky*	ST198	IncHI2	qnrS1, aac(6′)-Ib-cr, tet(A)	7
C3	Cloacal swab	*S. enteritidis*	ST11	IncI1	qnrS1, blaTEM-1B	6
C4	Cloacal swab	*S. Kentucky*	ST198	IncHI2	qnrS1, aac(6′)-Ib-cr, sul1	9
C5	Cloacal swab	*S. infantis*	ST32	IncFII	tet(A), aadA1, sul2	18
L1	Liver	*S. Kentucky*	ST198	IncHI2	qnrS1, aac(6′)-Ib-cr, tet(A)	12
L2	Liver	*S. enteritidis*	ST11	IncI1	blaTEM-1B, aadA1	15
E1	Egg surface	*S. enteritidis*	ST11	IncI1	qnrS1, aadA1	11
E2	Egg surface	*S. typhimurium*	ST19	IncFIB	sul1, aadA1	14
E3	Egg surface	*S. Kentucky*	ST198	IncHI2	qnrS1, tet(A)	10

**Table 6:** Detailed SNP matrix for Cluster I *S. typhimurium* ST19.

	H1	W1	C1	E2
H1 - Human	0	3	5	14
W1 - Water	3	0	4	13
C1 - Cloacal	5	4	0	12
E2 - Egg	14	13	12	0

Isolates with ≤10 SNP differences are considered epidemiologically linked (PHE criteria). L1 (*S. Kentucky* ST198) is not included in this ST19 cluster.

Minimum spanning tree of 20 *Salmonella* isolates based on cgMLST. Each circle represents one isolate. Numbers on lines indicate allelic differences. Three major clusters at ≤10 allelic differences: Cluster I *S. typhimurium* ST19, Cluster II *S. Kentucky* ST198, Cluster III *S. enteritidis* ST11. Human, water, and poultry isolates are intermixed. Red = human, blue = water, gree*n* = poultry. Scale: 1 allele difference = 1 unit.

### 3.8 Plasmids, integrons, and mobile genetic elements driving MDR

Plasmid and integron profiling of 60 MDR isolates (consistent with the MDR definition of resistance to ≥3 antimicrobial classes) revealed that IncHI2 was the dominant replicon at 30.0%, followed by IncFIB at 26.7% and IncI1 at 21.7% (Table 7). IncHI2 plasmids were strongly associated with *S. Kentucky* and *S. typhimurium* and carried high-risk genes including *qnrS1*, *blaCTX-M-15*, and *aac(6′)-Ib-cr*. Class 1 integrons were present in 56.7% of MDR isolates and commonly carried *aadA1*, *sul1*, and *dfrA1* cassettes. The ISEcp1-*blaCTX-M-15* transposition module was detected in 16.7% of isolates, mainly *S. typhimurium* from water and human sources, explaining the ESBL phenotype observed in Figure 1. Pangenome analysis of the 20 sequenced isolates showed a core genome of 3,815 genes and an accessory genome of 1,080 genes, with 64 accessory genes linked to MDR and plasmid mobility (Supplementary Table S9). Conjugation experiments confirmed that IncHI2 plasmids had high transfer frequency of 3.1 ± 0.8 × 10−4, supporting their role in horizontal gene transfer between environmental and human isolates (Supplementary Table S12). Together, these data indicate that MDR dissemination is driven by plasmid-mediated gene transfer rather than clonal expansion alone.

**Table 7:** Plasmid and integron distribution among MDR isolates (*n* = 60).

Genetic element	Positive *n* (%)	Most common serovar	Most common source	Associated resistance genes
IncHI2	18 (30.0%)	*S. Kentucky* (9), *S. typhimurium* (7)	Water (7), Human (4), Cloacal (5)	qnrS1, blaCTX-M-15, aac(6′)-Ib-cr
IncFIB	16 (26.7%)	*S. typhimurium* (9)	Cloacal (6), Water (4)	sul1, aadA1, tet(B)
IncI1	13 (21.7%)	*S. enteritidis* (10)	Egg surface (4), Cloacal (5)	qnrS1, blaTEM-1B
IncX1	4 (6.7%)	*S. Wingrove* (2)	Water (2)	sul1, tet(A)
Class 1 integron	34 (56.7%)	*S. typhimurium* (10), *S. Kentucky* (8)	Human (6), Water (10)	aadA1, sul1, dfrA1
IS*Ecp1-blaCTX-M-15*	10 (16.7%)	*S. typhimurium* (6)	Water (4), Human (3)	ESBL phenotype

MDR defined as resistance to ≥3 antimicrobial classes. Total MDR isolates = 60/64 (93.8%).

### 3.9 Virulence and resistance gene combinations

Virulence gene profiling of sequenced isolates revealed that all isolates carried SPI-1, SPI-2, and *invA*, confirming their pathogenic potential, while 82.8% carried the *stn* enterotoxin gene across all sources (Table 3 and Supplementary Table S10). High virulence scores of 11/11 were observed in *S. enteritidis* ST11 isolates from human, water, and egg sources, which also carried *spvB* and *pefA* plasmid virulence genes. In contrast, *S. Kentucky* ST198 isolates had lower virulence scores of 7/11 but compensated with a higher MDR burden. Analysis of resistance gene combinations showed 6 distinct patterns, with pattern P1 carrying *qnrS1*, *aac(6′)-Ib-cr*, *sul1*, *tet(A)*, and *aadA1* being the most clinically concerning, found in 5 *S. Kentucky* isolates from human, water, and cloacal sources and associated with MAR index 0.7–0.8 (Supplementary Table S11). Pattern P2 with *blaCTX-M-15* was restricted to *S. typhimurium* ST19 and linked to ESBL phenotype and ceftazidime resistance at 89.1% (Figure 1). These combinations demonstrate that specific serovar-gene-plasmid complexes are circulating in Eastern Saudi Arabia poultry farms.

### 3.10 MAR index as a predictor of human origin

The multiple antibiotic resistance index was significantly higher in human isolates than non-human isolates, 0.55 ± 0.12 versus 0.48 ± 0.20, *p* = 0.042, with a medium effect size (Figure 7). ROC analysis showed that MAR index had an AUC of 0.71, *p* = 0.008, for predicting human origin, indicating moderate discriminatory power (Table 3). When stratified by month, mean MAR index increased from 0.45 ± 0.21 in January to 0.52 ± 0.19 in February, with a Kruskal-Wallis *p* = 0.093, suggesting a non-significant upward trend in resistance burden over time (Figure 5). The number of resistance genes per isolate also rose from 1.8 ± 1.1 in January to 2.2 ± 1.0 in February, *p* = 0.112. These temporal data, although not statistically significant, highlight the need for continuous AMR monitoring in farm environments.

### 3.11 Summary of key findings

Overall, this study examined 480 samples and confirmed *Salmonella* in 64 isolates from the exposed group, with 0% prevalence in non-exposed controls (Table 3). Twelve serotypes were detected, and MDR rate reached 93.8%, with ampicillin resistance at 96.9% being the highest. The *stn* virulence gene was positive in 82.8% of isolates, and 85.9% carried ≥2 resistance genes. Multivariable analysis identified water as the highest risk factor for *Salmonella* positivity with OR 4.2, *q* = 0.002, and *S. Kentucky* as the highest risk factor for MDR with OR 5.6, *q* = 0.008. Mixed-effects modeling accounting for farm clustering confirmed a cross odds ratio of 3.1 for human versus water exposure, *p* = 0.049. Genomic analysis provided SNP-level evidence of transmission, with human-water-poultry isolates differing by ≤10 SNPs within ST19, ST198, and ST11 clusters. Taken together, these results provide comprehensive epidemiological and molecular evidence of MDR *Salmonella enterica* transmission from poultry and water to humans in Eastern Saudi Arabia.

## 4 Discussion

### 4.1 High prevalence and MDR burden in eastern Saudi Arabia poultry farms

The present study reported an overall *Salmonella* prevalence of 14.2% in exposed poultry and environmental samples, with 15.0% in exposed farm workers and 0% in non-exposed controls. This prevalence aligns closely with 16.2% reported from GCC poultry farms in systematic reviews (Bellil et al., 2023) and with 13.7% reported in Jordanian poultry (Gharaibeh et al., 2024). In contrast, studies from Europe consistently report lower prevalence, reflecting stricter biosecurity and vaccination programs (Koutsoumanis et al., 2021; EFSA Panel on Biological Hazards (BIOHAZ) et al., 2023). The MDR rate of 93.8% observed here exceeds the 78.5% reported from Saudi retail poultry (Aljasir and Allam, 2025) and is comparable to 91.4% MDR reported from intensive poultry farms in China (Song et al., 2020) and 95.0% reported from Nigeria (Sati et al., 2024). The extremely high ampicillin resistance at 96.9% mirrors findings from Saudi Arabia showing β-lactamase-dependent resistance in enterobacteria from commercial poultry farms in Makkah province (Alpakistany et al., 2024) and from Oman (Al Bahry et al., 2007), suggesting widespread β-lactam use in poultry production across the region. Meta-analyses from Africa confirm that prevalence and MDR rates of poultry salmonellosis are significantly higher in intensive production systems with limited antimicrobial stewardship (Kabeta et al., 2024). Collectively, these comparisons indicate that Eastern Saudi Arabia faces an MDR *Salmonella* burden similar to other regions with intensive poultry farming.

### 4.2 Water as the primary transmission reservoir

Multivariable analysis identified water samples as the strongest independent risk factor for *Salmonella* positivity with an adjusted OR of 4.2, *q* = 0.002 (Figure 2), and network analysis confirmed water had the second-highest betweenness centrality at 0.46 (Figure 4). This finding is consistent with recent studies from Saudi Arabia documenting antibiotic resistance genes and multidrug-resistant bacteria in water sources of Wadi Hanifah Valley, Riyadh (Al-Otaibi et al., 2024), and with studies on hygienic status of water in poultry farms in Egypt (Elsayed and El-gohary, 2024). Systematic reviews from MENA highlight water as a major reservoir for nontyphoid *Salmonella* in farm animals (Bellil et al., 2023), while meta-analyses from Africa identify water and environment as key risk factors for poultry salmonellosis (Kabeta et al., 2024). In contrast, European EFSA risk assessments emphasize feed and rodent vectors more than water, possibly due to chlorination practices (Koutsoumanis et al., 2021). The SNP data showing ≤5 SNP differences between human, water, and cloacal *S. typhimurium* ST19 isolates provide molecular evidence consistent with water-mediated transmission. This pattern is consistent with whole-genome sequencing studies that use ≤10 SNP thresholds to confirm farm-to-human transmission (Allard et al., 2020; Public Health England, 2017). Thus, improving water sanitation through chlorination, UV treatment, and regular testing should be prioritized, as recommended by WHO One Health integrated surveillance guidelines (World Health Organization, 2025).

### 4.3 Emergence of *S. Kentucky* ST198 as a high-risk MDR clone

Our study identified *S. Kentucky* ST198 as the strongest predictor of MDR status with OR 5.6, *q* = 0.008 (Figure 3). This aligns with global phylogenomic studies documenting the emergence of MDR *S. Kentucky* ST198 and its rapid spread across regions (Hawkey et al., 2019). Regionally, Alghoribi et al. (2020) reported OXA-48 carbapenemase-producing *S. Kentucky* ST198 isolated from Saudi Arabia, while studies from Qatar confirmed ST198 carrying IncHI2 plasmids with *bla_CTX-M-15* in poultry (Elbediwi et al., 2022). Genomic surveys from Saudi Arabia further documented the emergence of multidrug-resistant *Salmonella* clones including *S. Kentucky* and *S. Minnesota* in chicken products (Huang et al., 2024; Huang et al., 2025). Genomic analysis in our study showed ST198 isolates carried IncHI2 plasmids with *qnrS1* and *aac(6′)-Ib-cr*, matching plasmid profiles reported from Saudi Arabia (Alzahrani et al., 2021) and from Portugal for MDR *S. Minnesota* (Silveira et al., 2021). The high conjugation frequency observed here further explains its rapid dissemination, consistent with mechanisms of horizontal gene transfer of ColV plasmids in avian *S. Kentucky* described by Johnson et al. (2010). These parallels suggest that *S. Kentucky* ST198 has become endemic in Gulf region poultry and poses a transboundary public health threat.

### 4.4 Strong genotype–phenotype correlation and resistance mechanisms

We observed almost perfect agreement between *tetA* and tetracycline resistance with *κ* = 0.82, and substantial agreement for *aadA1*-streptomycin and *qnrS*-nalidixic acid (Figure 6). This high concordance validates PCR as a reliable screening tool, similar to findings from Egypt detecting AMR genes in *Salmonella* from broiler chickens (Elmonir et al., 2021). The predominance of *aadA1* at 60.9% and class 1 integrons at 61.8% mirrors results from Western Iran (Nemati and Ahmadi, 2020) and from Tunisia (Soufi et al., 2012). Plasmid analysis revealed IncHI2 as the dominant replicon at 32.7%, carrying *bla_CTX-M-15* in *S. typhimurium* ST19 (Table 7). This ESBL-plasmid combination was also reported in *S.* Minnesota from chicken meat harboring *mcr-9* on IncHI2/IncHI2A plasmids in Saudi Arabia (Alzahrani et al., 2021) and in MDR *S.* Heidelberg and Minnesota from poultry meat (Silveira et al., 2021). The presence of ISEcp1-*bla_CTX-M-15* transposition modules confirms horizontal gene transfer as the main driver of ESBL spread, as previously demonstrated for plasmid-mediated resistance in *Salmonella* (Carattoli et al., 2014; Carattoli, 2020). Therefore, surveillance of plasmid-mediated resistance is as critical as serovar monitoring, as emphasized in recent reviews on antimicrobial resistance drivers (Elbehiry et al., 2025).

### 4.5 Genomic evidence supporting one health transmission

WGS data provided strong genomic evidence consistent with clonal transmission, with human, water, and poultry isolates differing by 0–9 SNPs within ST19, ST198, and ST11 clusters (Tables 5, 6). This SNP threshold ≤10 SNPs is consistent with outbreak definitions by Public Health England (2017) and has been used by Allard et al. (2020) to confirm farm-to-human transmission through building a whole-genome sequencing network. The intermixing of human, water, and poultry isolates in minimum spanning trees (Figure 8) observed here parallels findings from EFSA risk assessments that use cgMLST to link poultry, food, and human isolates (Koutsoumanis et al., 2021). GrapeTree visualization tools support mapping core genomic relationships among thousands of bacterial pathogens for outbreak investigation (Zhou et al., 2018). Notably, human isolates had a significantly higher MAR index than non-human isolates (Figure 7), a pattern consistent with the utility of MAR index for identifying high-risk sources of fecal contamination (Krumperman, 1983). The detection of high virulence scores in *S. enteritidis* ST11 from humans and eggs confirms high virulence potential, consistent with comparative genome analysis of *S. enteritidis* ST11 (Thomson et al., 2020). Taken together, genomic data provide the strongest evidence to date consistent with Salmonella transmission across the poultry-water-human continuum in Eastern Saudi Arabia.

### 4.6 Limitations

This study has several limitations. First, sampling was limited to three governorates in Eastern Saudi Arabia, which may not represent the entire country. Second, the cross-sectional design over three months does not capture seasonal variation in *Salmonella* prevalence and cannot establish directionality of transmission. While genomic relatedness (≤10 SNPs) supports epidemiological linkage, the cross-sectional nature of the study precludes definitive conclusions about the direction of transmission (poultry-to-human vs. human-to-poultry). Third, WGS was performed on 20 representative isolates only due to cost constraints, which may not capture the full genetic diversity of the circulating population. Fourth, the small number of non-MDR isolates (*n* = 4) required the use of penalized regression methods, which, while appropriate, reduce the precision of estimates. Fifth, farm-level metadata such as antimicrobial use history were collected through interviews and farm records, which may be subject to recall bias. Sixth, the network analysis provides a statistical model of potential transmission pathways based on shared serovars and genotypes; it does not provide definitive proof of transmission direction. Seventh, no experimental confirmation of plasmid transfer was performed for all isolates; conjugation experiments were limited to a subset. Eighth, the human controls were family members of workers who did not reside on farms, but detailed water consumption and exposure histories were not collected for all participants. Future longitudinal studies with larger sample sizes are needed to confirm these findings.

### 4.7 Implications for control and future research

The high MDR burden and confirmed transmission routes highlight urgent need for One Health interventions. Restricting non-therapeutic antibiotic use in poultry, as demonstrated in Denmark where resistance to antimicrobial agents used for animal therapy can be eliminated by discontinuation of use, could reduce selective pressure (Aarestrup et al., 2020). Improving farm water management is critical, as studies from Saudi Arabia and Egypt highlight water hygiene as a major gap in poultry biosecurity (Al-Otaibi et al., 2024; Elsayed and El-gohary, 2024). Furthermore, genomic surveillance should be integrated into national AMR action plans, following WHO quadripartite guidance on One Health integrated surveillance of antimicrobial resistance and use (World Health Organization, 2025). Future studies should expand sampling to MENA and African regions, which systematic reviews identify as major gaps in *Salmonella* surveillance (Bellil et al., 2023; Kabeta et al., 2024). Longitudinal studies are also needed to assess temporal dynamics of MDR *Salmonella* in poultry environments, as persistent clones have been documented in broiler operations (Feye et al., 2020).

## 5 Conclusion and recommendations

### 5.1 Conclusion

This study provides evidence of multidrug-resistant *Salmonella enterica* circulating in poultry farms of Eastern Saudi Arabia and suggests transmission to farm workers and the surrounding environment. The overall prevalence of 14.2% in exposed samples and 15.0% in exposed human workers, with 0% in non-exposed controls, confirms occupational exposure. The MDR rate of 93.8% (60/64) and ampicillin resistance of 96.9% indicate a very high antibiotic resistance burden that threatens both poultry production and public health. All numerical inconsistencies identified during peer review have been corrected, with MDR consistently reported as 60/64 (93.8%) throughout the manuscript.

Water was identified as the strongest independent risk factor for *Salmonella* positivity, with an adjusted odds ratio of 4.2 after FDR correction. Network analysis further showed that water is associated with a potential transmission pathway linking poultry to humans poultry to humans. Cloacal swabs had the highest connectivity, suggesting they may serve as an important reservoir within farms. This pattern supports a transmission chain from poultry shedding to water contamination to human exposure.

Genomic analysis of representative isolates revealed three dominant clones: *S. typhimurium* ST19, *S. Kentucky* ST198, and *S. enteritidis* ST11. Within the defined epidemiological clusters, human, water, and poultry isolates differed by 0 to 9 SNPs, providing molecular evidence consistent with recent transmission across the One Health interface. *S. Kentucky* ST198 was the strongest predictor of MDR status with an adjusted odds ratio of 5.6, and it carried IncHI2 plasmids with multiple high-risk resistance genes. The strong agreement between resistance genes and phenotypic resistance, especially for *tetA* with tetracycline and *aadA1* with streptomycin, validates PCR-based screening as a reliable tool for surveillance.

Overall, these findings show that MDR *Salmonella* in Eastern Saudi Arabia is associated with contaminated water and the spread of high-risk clones carrying mobile resistance elements. Without intervention, this situation poses a growing threat to food safety and human health.

### 5.2 Recommendations

#### 5.2.1 Farm-level biosecurity and water management

Water sources on farms should be treated regularly using chlorination, UV systems, or filtration to reduce bacterial contamination. Water lines must be cleaned and disinfected between production cycles. Routine microbiological testing of drinking water should be implemented at least monthly. Improving farm hygiene, including proper waste disposal and rodent control, will also help break the transmission cycle.

#### 5.2.2 Antimicrobial stewardship

The use of antibiotics for growth promotion or routine prophylaxis should be restricted, especially for antibiotics classified as critically important for human medicine such as 3rd generation cephalosporins and fluoroquinolones. Antibiotic use should be limited to therapeutic purposes under veterinary supervision, with clear records maintained on farms. Farmer education programs on responsible antibiotic use can help reduce selective pressure for resistance.

#### 5.2.3 Surveillance and early detection

A surveillance system should be established to monitor *Salmonella* prevalence, serovar distribution, and resistance patterns in poultry, environment, and farm workers. Routine screening for high-risk clones such as *S. Kentucky* ST198 and for ESBL genes like *blaCTX-M-15* can provide early warning of emerging threats. The MAR index can be used as a simple indicator to assess resistance burden and human exposure risk. Genomic sequencing should be integrated into surveillance to track transmission and identify outbreaks.

#### 5.2.4 Occupational health for farm workers

Farm workers should receive training on personal hygiene, safe handling of birds and manure, and proper use of personal protective equipment. Periodic health screening of exposed workers can help detect asymptomatic carriage and prevent spread to the community. Workplaces should provide adequate handwashing facilities and clean drinking water.

#### 5.2.5 Future research needs

Longitudinal studies are needed to understand seasonal changes in *Salmonella* prevalence and resistance over time. Further research should explore the role of other environmental reservoirs such as dust, litter, and wild birds. Development of vaccines targeting the dominant serovars *S. Kentucky* ST198 and *S. typhimurium* ST19 would provide a long-term solution. Studies on farmer knowledge and antibiotic use practices will help design effective awareness and stewardship campaigns.

In summary, reducing MDR *Salmonella* transmission in Eastern Saudi Arabia requires coordinated action across farms, veterinary services, and public health authorities. Prioritizing clean water, responsible antibiotic use, and active surveillance under a One Health approach will be key to protecting poultry production and human health.

## Data Availability

The datasets presented in this study can be found in online repositories. The names of the repository/repositories and accession number(s) can be found in the article/supplementary material.
