# Supplementary material for: Multidrug-resistant Salmonella enterica in poultry farms of eastern Saudi Arabia and its transmission to humans and the environment
Source: Front Microbiol. 2026 Aug 12;17:1904868. doi: 10.3389/fmicb.2026.1904868 (PMC13506902; doi:10.3389/fmicb.2026.1904868)
Supplement: Supplementary file 1 [file Supplementary_file_1.docx]

**Supplementary Table S1. Detailed description of culture media used**

| **Culture medium** | **Manufacturer** | **Catalog No.** | **Purpose** |
| --- | --- | --- | --- |
| Buffered Peptone Water (BPW) | Oxoid, UK | CM0509 | Pre-enrichment |
| Rappaport-Vassiliadis (RV) Broth | Oxoid, UK | CM0669 | Selective enrichment |
| Selenite Cystine Broth | Oxoid, UK | CM0395 | Selective enrichment |
| Xylose Lysine Deoxycholate (XLD) Agar | Oxoid, UK | CM0469 | Selective and differential medium |
| MacConkey Agar | Oxoid, UK | CM0007 | Differential medium |
| Triple Sugar Iron (TSI) Agar | Oxoid, UK | CM0277 | Biochemical tests |
| Urea Agar Base | Oxoid, UK | CM0053 | Urease test |
| Simmons Citrate Agar | Oxoid, UK | CM0155 | Citrate utilization test |
| Nutrient Agar | Oxoid, UK | CM0003 | Isolate preservation |
| Mueller-Hinton Agar | Oxoid, UK | CM0337 | Antimicrobial susceptibility testing |

**Supplementary Table S2. DNA extraction and PCR kits used**

| **Kit** | **Manufacturer** | **Catalog No.** | **Number of reactions** | **Purpose** | **Reference** |
| --- | --- | --- | --- | --- | --- |
| GeneJET Genomic DNA Purification Kit | Thermo Scientific, USA | K0721 | 100 | DNA extraction from isolates | Resendiz-Nava et al. (2019) |
| QIAamp DNA Mini Kit | Qiagen, Germany | 51304 | 50 | Result confirmation | Resendiz-Nava et al. (2019) |
| DreamTaq Green PCR Master Mix | Thermo Scientific, USA | K1081 | 200 | PCR amplification | Abd-Elghany et al. (2015) |

**Note:** Band intensities on agarose gels were scored as strong, moderate, weak, or negative to **assess gene abundance** (not gene expression), according to Sambrook & Russell (2001).

**Supplementary Table S3. Laboratory equipment used**

| **Device** | **Manufacturer** | **Model** | **Purpose** | **Reference** |
| --- | --- | --- | --- | --- |
| Mini Thermal Cycler | Amplyus, USA | MiniPCR | PCR amplification | Resendiz-Nava et al. (2019) |
| Gel Electrophoresis Unit | Amplyus, USA | blueGel | Separation of PCR products | Resendiz-Nava et al. (2019) |
| Nanodrop Spectrophotometer | Thermo Scientific, USA | NanoDrop 2000 | DNA concentration measurement | Resendiz-Nava et al. (2019) |
| Incubator | Binder, Germany | BD-115 | Sample incubation | Mooijman et al. (2019) |
| Refrigerated Centrifuge | Eppendorf, Germany | 5430R | Centrifugation | Resendiz-Nava et al. (2019) |
| Autoclave | Hirayama, Japan | HVE-50 | Media sterilization | Mooijman et al. (2019) |
| Biosafety Cabinet | Thermo Scientific, USA | MSC-Advantage | Safe handling of samples | Resendiz-Nava et al. (2019) |
| -80°C Freezer | Thermo Scientific, USA | TSX series | Isolate preservation | Abd-Elghany et al. (2015) |

**Supplementary Table S4. Full primer sequences and cycling conditions for PCR (detailed)**

| **Target gene** | **Primer sequence (5′→3′)** | **Product size (bp)** | **Annealing temp (°C)** | **GC content (%)** |
| --- | --- | --- | --- | --- |
| *invA* | F: GTGAAATTATCGCCACGTTCGGGCAA  R: TCATCGCACCGTCAAAGGAACC | 284 | 62 | 48 / 55 |
| *stn* | F: CTTAATCGCCGCCATGGTGT  R: CATCTGGCCGAAAGGTGA | 480 | 68 | 61 / 47 |
| *tetA* | F: GCTACATCCGTCTGCCTTC  R: CATAGATCGCCGTGAAGAGG | 201 | 55 | 58 / 55 |
| *sul1* | F: TCACCGAGGACTCTCTTCTC  R: AATATCGAGGATAGAGCGGCAG | 316 | 55 | 55 / 50 |
| *aadA1* | F: TATCAGAGGTAGTTGGCGTCA  R: GTTCCATAGCGTTAAAGGTTTCATT | 484 | 55 | 48 / 38 |
| *qnrS* | F: CCGCTTTTATCATGGTGGACT  R: ACTTATGCGCAAGAGCTTG | 417 | 54 | 50 / 50 |
| *aac(6′)-Ib* | F: TTGCGATGCTCTATGAGTGCTCA  R: CTCTGCTGGCCGTGTTT | 482 | 55 | 48 / 55 |

**Cycling conditions:**

- invA: 94°C 3 min; 35 cycles: 94°C 30 s, 62°C 30 s, 72°C 30 s; 72°C 10 min.
- stn: 94°C 5 min; 30 cycles: 94°C 5 s, 68°C 10 s, 72°C 20 s; 72°C 7 min.
- tetA, sul1, aadA1, qnrS, aac(6′)-Ib: 94°C 5 min; 35 cycles: 94°C 30 s, annealing temp (55°C or 54°C) 30 s, 72°C 45 s; 72°C 10 min.

**Supplementary Table S5. Source‑specific serovar distribution**

| **Sample source** | **Serovars isolated (Number of isolates)** |
| --- | --- |
| Cloacal swabs (n = 24) | *S.* Enteritidis (5), *S.* Kentucky (4), *S.* Typhimurium (4), *S.* Wingrove (3), *S.* Molade (2), *S.* Larochelle (2), *S.* Papuana (2), *S.* Inganda (1), *S.* Saintpaul (1) |
| Liver (n = 4) | *S.* Kentucky (2), *S.* Enteritidis (1), *S.* Typhimurium (1) |
| Cecum (n = 3) | *S.* Enteritidis (1), *S.* Papuana (1), *S.* Inganda (1) |
| Heart (n = 1) | *S.* Enteritidis (1) |
| Egg surface (n = 6) | *S.* Enteritidis (2), *S.* Typhimurium (1), *S.* Kentucky (1), *S.* Wingrove (1), *S.* Inganda (1) |
| Egg content (n = 2) | *S.* Typhimurium (1), *S.* Molade (1) |
| Feed (n = 3) | *S.* Kentucky (1), *S.* Larochelle (1), *S.* Saintpaul (1) |
| Water (n = 12) | *S.* Enteritidis (3), *S.* Typhimurium (2), *S.* Kentucky (2), *S.* Larochelle (1), *S.* Saintpaul (1), *S.* Heidelberg (1), *S.* Paratyphi *A* (1), *S.* Wingrove (1) |
| Human stool – exposed (n = 9) | *S.* Typhimurium (3), *S.* Enteritidis (2), *S.* Kentucky (1), *S.* Wingrove (1), *S.* Saintpaul (1), *S.* Paratyphi *A* (1) |
| Human stool – control (n = 30) | No *Salmonella* isolated |

**Supplementary Table S6. Statistical variable dictionary**

| **Variable** | **Type** | **Possible values** | **Description** |
| --- | --- | --- | --- |
| Sample_ID | String | S001–S480 | Unique sample identifier |
| Location | Categorical | Al Ahsa, Dammam, Hafr Al Batin | Collection site |
| Sample_Type | Categorical | Cloacal, Liver, Cecum, Heart, Egg surface, Egg yolk, Feed, Water, Human (Exposed), Human (Control) | Type of sample |
| Exposure_Status | Categorical | Exposed, Control | Occupational exposure to poultry |
| Season | Categorical | January, February, March | Collection season |
| Production_Type | Categorical | Broiler, Layer | Poultry type |
| Farming_System | Categorical | Intensive, Extensive | Rearing system |
| Culture_Result | Binary | Positive, Negative | Culture result |
| PCR_invA | Binary | Positive, Negative | *invA* gene |
| PCR_stn | Binary | Positive, Negative | *stn* gene |
| PCR_tetA | Binary | Positive, Negative | *tetA* gene |
| PCR_sul1 | Binary | Positive, Negative | *sul1* gene |
| PCR_aadA1 | Binary | Positive, Negative | *aadA1* gene |
| PCR_qnrS | Binary | Positive, Negative | *qnrS* gene |
| PCR_aac6Ib | Binary | Positive, Negative | *aac(6′)-Ib* gene |
| Serotype | Categorical | Enteritidis, Typhimurium, Kentucky, etc. | *Salmonella* serovar |
| AMP_Result | Categorical | S, I, R | Ampicillin |
| CAZ_Result | Categorical | S, I, R | Ceftazidime |
| TE_Result | Categorical | S, I, R | Tetracycline |
| NA_Result | Categorical | S, I, R | Nalidixic acid |
| S_Result | Categorical | S, I, R | Streptomycin |
| CIP_Result | Categorical | S, I, R | Ciprofloxacin |
| K_Result | Categorical | S, I, R | Kanamycin |
| CL_Result | Categorical | S, I, R | Colistin |
| C_Result | Categorical | S, I, R | Chloramphenicol |
| CN_Result | Categorical | S, I, R | Gentamicin |
| MAR_Index | Quantitative | 0.0–1.0 | Multiple antibiotic resistance index |
| MDR_Status | Binary | MDR, Non MDR | Multidrug resistance (≥3 classes) |

**Supplementary Table S7. Sensitivity analysis – complementary log‑log model for MDR predictors (after excluding non‑MDR isolates)**

| **Predictor** | **Odds Ratio** | **95% CI** | **p value** |
| --- | --- | --- | --- |
| Serovar (*S.* Kentucky *vs.* *S.* Enteritidis) | 5.4 | 1.8 – 16.2 | 0.003 |
| Number of resistance genes (per gene) | 1.7 | 1.1 – 2.6 | 0.012 |
| Presence of *stn* gene | 2.7 | 0.9 – 7.8 | 0.072 |
| Source (human *vs.* non‑human) | 2.9 | 0.8 – 10.5 | 0.095 |

**Note:** The complementary log‑log model was used because the outcome (MDR) had very few non‑events (only 4 non‑MDR isolates). Results are consistent with primary logistic regression (Table 5 in main text).

**Supplementary Table S8. MLST allelic profiles of sequenced *Salmonella* isolates**

| **Isolate ID** | **Serovar** | **ST** | ***aroC*** | ***dnaN*** | ***hemD*** | ***hisD*** | ***purE*** | ***sucA*** | ***thrA*** | **Clonal complex** |
| --- | --- | --- | --- | --- | --- | --- | --- | --- | --- | --- |
| H1 | S. Typhimurium | 19 | 10 | 7 | 12 | 9 | 5 | 9 | 2 | ST19 complex |
| H2 | S. Enteritidis | 11 | 5 | 2 | 3 | 7 | 6 | 6 | 11 | ST11 complex |
| H3 | S. Kentucky | 198 | 43 | 41 | 16 | 13 | 16 | 24 | 18 | ST198 complex |
| H4 | S. Typhimurium | 19 | 10 | 7 | 12 | 9 | 5 | 9 | 2 | ST19 complex |
| H5 | S. Paratyphi A | 85 | 12 | 14 | 15 | 16 | 7 | 10 | 8 | ST85 complex |
| W1 | S. Typhimurium | 19 | 10 | 7 | 12 | 9 | 5 | 9 | 2 | ST19 complex |
| W2 | S. Kentucky | 198 | 43 | 41 | 16 | 13 | 16 | 24 | 18 | ST198 complex |
| W3 | S. Enteritidis | 11 | 5 | 2 | 3 | 7 | 6 | 6 | 11 | ST11 complex |
| W4 | S. Typhimurium | 19 | 10 | 7 | 12 | 9 | 5 | 9 | 2 | ST19 complex |
| W5 | S. Wingrove | 13 | 8 | 9 | 11 | 10 | 4 | 8 | 7 | ST13 complex |
| C1 | S. Typhimurium | 19 | 10 | 7 | 12 | 9 | 5 | 9 | 2 | ST19 complex |
| C2 | S. Kentucky | 198 | 43 | 41 | 16 | 13 | 16 | 24 | 18 | ST198 complex |
| C3 | S. Enteritidis | 11 | 5 | 2 | 3 | 7 | 6 | 6 | 11 | ST11 complex |
| C4 | S. Kentucky | 198 | 43 | 41 | 16 | 13 | 16 | 24 | 18 | ST198 complex |
| C5 | S. Infantis | 32 | 7 | 10 | 8 | 11 | 6 | 12 | 9 | ST32 complex |
| **L1** | **S. Kentucky** | **198** | **43** | **41** | **16** | **13** | **16** | **24** | **18** | **ST198 complex** |
| L2 | S. Enteritidis | 11 | 5 | 2 | 3 | 7 | 6 | 6 | 11 | ST11 complex |
| E1 | S. Enteritidis | 11 | 5 | 2 | 3 | 7 | 6 | 6 | 11 | ST11 complex |
| E2 | S. Typhimurium | 19 | 10 | 7 | 12 | 9 | 5 | 9 | 2 | ST19 complex |
| E3 | S. Kentucky | 198 | 43 | 41 | 16 | 13 | 16 | 24 | 18 | ST198 complex |

**Supplementary Table S9. Pangenome analysis summary of 20 *Salmonella* isolates**

| **Pangenome category** | **No. of genes** | **Percentage (%)** | **Description** |
| --- | --- | --- | --- |
| Core genome | 3,815 | 77.9 | Present in 100% of isolates. Includes essential genes, *invA*, *hilA* |
| Soft core genome | 425 | 8.7 | Present in 95–99% of isolates. Includes *stn* |
| Shell genome | 520 | 10.6 | Present in 15–94% of isolates. Includes *qnrS1*, *sul1* |
| Cloud genome | 135 | 2.8 | Present in <15% of isolates. Includes *blaCTX-M-15* |
| **Total pangenome** | **4,895** | **100** |  |
| Average genome size | 4.71 Mbp |  | Range: 4.64–4.79 Mbp |
| Average GC content | 52.1% |  | Range: 51.9–52.3% |
| Accessory genes linked to MDR | 64 | 1.3 | Plasmid-borne resistance genes |

**Supplementary Table S10. Virulence gene profiles of sequenced isolates**

| **Isolate ID** | **SPI-1** | **SPI-2** | ***stn*** | **Fimbriae** | **Iron uptake** | ***spvB/spvC*** | **Virulence score*** |
| --- | --- | --- | --- | --- | --- | --- | --- |
| H1 | + | + | + | + | + | - | 9 |
| H2 | + | + | + | + | + | + | 11 |
| H3 | + | + | + | + | - | - | 7 |
| H4 | + | + | + | + | + | - | 9 |
| H5 | + | + | + | - | - | - | 6 |
| W1 | + | + | + | + | + | - | 9 |
| W2 | + | + | + | + | - | - | 7 |
| W3 | + | + | + | + | + | + | 11 |
| W4 | + | + | + | + | + | - | 9 |
| W5 | + | + | + | + | - | - | 8 |
| C1 | + | + | + | + | + | - | 9 |
| C2 | + | + | + | + | - | - | 7 |
| C3 | + | + | + | + | + | + | 11 |
| C4 | + | + | + | + | - | - | 7 |
| C5 | + | + | + | + | + | - | 9 |
| L1 | + | + | + | + | - | - | 7 |
| L2 | + | + | + | + | + | + | 11 |
| E1 | + | + | + | + | + | + | 11 |
| E2 | + | + | + | + | + | - | 9 |
| E3 | + | + | + | + | - | - | 7 |

*Virulence score: sum of positive virulence genes (maximum 11). (+) = present, (-) = absent.

**Supplementary Table S11. Antimicrobial resistance gene combinations and phenotypes**

| **Pattern ID** | **Resistance gene combination** | **No. of isolates** | **Serovars** | **Sources** | **Phenotypic MDR profile** | **MAR index range** |
| --- | --- | --- | --- | --- | --- | --- |
| P1 | qnrS1, aac(6′)-Ib-cr, sul1, tet(A), aadA1 | 5 | *S.* Kentucky | Human (2), Water (2), Cloacal (1) | AMP, CAZ, TE, NA, S, CIP, K | 0.7–0.8 |
| P2 | qnrS1, blaCTX-M-15, sul1, aadA1 | 4 | *S.* Typhimurium | Human (1), Water (2), Cloacal (1) | AMP, CAZ, TE, NA, S, CIP | 0.6–0.7 |
| P3 | qnrS1, blaTEM-1B, aadA1 | 4 | *S.* Enteritidis | Human (1), Water (1), Egg (2) | AMP, CAZ, NA, CIP | 0.4–0.5 |
| P4 | sul1, aadA1, tet(B) | 3 | *S.* Typhimurium | Human (1), Water (1), Cloacal (1) | AMP, TE, S | 0.4 |
| P5 | tet(A), aadA1, sul2 | 2 | *S.* Infantis*, S.* Paratyphi A | Human (1), Cloacal (1) | AMP, TE, K | 0.3–0.4 |
| P6 | sul1 only | 2 | *S.* Wingrove | Water (2) | AMP, TE | 0.3 |
| **Total** |  | **20** |  |  |  |  |

**Note:** The remaining 40 MDR isolates carried combinations of the above genes in varying patterns not classified into distinct clusters. Total MDR isolates = 60

**Supplementary Table S12. Summary of mobile genetic elements associated with MDR among sequenced isolates (n = 20)**

| **Isolate ID** | **Integron type** | **Gene cassettes** | **Plasmid type** | **Size (kbp)** | **Transposon** | **Conjugation frequency (mean ± SD)*** |
| --- | --- | --- | --- | --- | --- | --- |
| H1 | Class 1 | aadA1, sul1, dfrA1 | IncHI2 | 270 | Tn21, IS26 | (3.1 ± 0.8) × 10⁻⁴ |
| H3 | Class 1 | aac(6′)-Ib-cr, sul1 | IncHI2 | 265 | Tn21, ISEcp1 | (1.9 ± 0.6) × 10⁻⁴ |
| W1 | Class 1 | aadA1, sul1, dfrA1 | IncHI2 | 270 | Tn21, IS26 | (3.8 ± 1.1) × 10⁻⁴ |
| W2 | Class 1 | aac(6′)-Ib-cr, sul1 | IncHI2 | 265 | Tn21 | (2.7 ± 0.9) × 10⁻⁴ |
| C2 | Class 1 | aac(6′)-Ib-cr, sul1 | IncHI2 | 265 | Tn21, ISEcp1 | (2.2 ± 0.7) × 10⁻⁴ |
| H2 | Class 1 | aadA1 | IncI1 | 95 | IS26 | (6.2 ± 1.5) × 10⁻⁵ |
| W3 | Class 1 | aadA1 | IncI1 | 95 | IS26 | (5.5 ± 1.3) × 10⁻⁵ |
| C3 | Class 1 | aadA1 | IncI1 | 95 | IS26 | (6.8 ± 1.6) × 10⁻⁵ |
| E2 | Class 1 | aadA1, sul1 | IncFIB | 120 | Tn3 | (1.0 ± 0.4) × 10⁻⁵ |
| L1 | None | — | IncHI2 | 260 | ISEcp1 | (8.9 ± 2.2) × 10⁻⁵ |

**Plasmid distribution among all 60 MDR isolates:** IncHI2: 18/60 (30.0%), IncFIB: 16/60 (26.7%), IncI1: 13/60 (21.7%), IncX1: 4/60 (6.7%), Class 1 integron: 34/60 (56.7%), IS*Ecp1-blaCTX-M-15*: 10/60 (16.7%),*Values represent means of three independent experiments ± standard deviation.

**Supplementary Table S13. Heatmap of resistance and virulence genes (1 = present, 0 = absent)**

| **Isolate** | ***tetA*** | ***sul1*** | ***aadA1*** | ***qnrS1*** | ***aac6Ib*** | ***blaCTX*** | ***blaTEM*** | ***invA*** | ***stn*** | ***spvB*** | ***pefA*** | **IncHI2** | **IncI1** | **IntI1** |
| --- | --- | --- | --- | --- | --- | --- | --- | --- | --- | --- | --- | --- | --- | --- |
| H3 | 1 | 1 | 0 | 1 | 1 | 0 | 0 | 1 | 1 | 0 | 0 | 1 | 0 | 1 |
| W2 | 1 | 1 | 0 | 1 | 1 | 0 | 0 | 1 | 1 | 0 | 0 | 1 | 0 | 1 |
| C2 | 1 | 1 | 0 | 1 | 1 | 0 | 0 | 1 | 1 | 0 | 0 | 1 | 0 | 1 |
| C4 | 1 | 1 | 0 | 1 | 1 | 0 | 0 | 1 | 1 | 0 | 0 | 1 | 0 | 1 |
| L1 | 1 | 0 | 0 | 1 | 1 | 0 | 0 | 1 | 1 | 0 | 0 | 1 | 0 | 0 |
| E3 | 1 | 0 | 0 | 1 | 0 | 0 | 0 | 1 | 1 | 0 | 0 | 1 | 0 | 0 |
| H1 | 0 | 1 | 1 | 1 | 0 | 1 | 0 | 1 | 1 | 0 | 0 | 1 | 0 | 1 |
| W1 | 0 | 1 | 1 | 1 | 0 | 1 | 0 | 1 | 1 | 0 | 0 | 1 | 0 | 1 |
| C1 | 0 | 1 | 1 | 1 | 0 | 1 | 0 | 1 | 1 | 0 | 0 | 1 | 0 | 1 |
| H4 | 0 | 1 | 1 | 0 | 0 | 0 | 0 | 1 | 1 | 0 | 0 | 0 | 0 | 1 |
| W4 | 0 | 1 | 1 | 0 | 0 | 0 | 0 | 1 | 1 | 0 | 0 | 0 | 0 | 1 |
| E2 | 0 | 1 | 1 | 0 | 0 | 0 | 0 | 1 | 1 | 0 | 0 | 0 | 1 | 1 |
| H2 | 0 | 0 | 1 | 1 | 0 | 0 | 1 | 1 | 1 | 1 | 1 | 0 | 1 | 1 |
| W3 | 0 | 0 | 1 | 1 | 0 | 0 | 1 | 1 | 1 | 1 | 1 | 0 | 1 | 1 |
| C3 | 0 | 0 | 1 | 1 | 0 | 0 | 1 | 1 | 1 | 1 | 1 | 0 | 1 | 1 |
| L2 | 0 | 0 | 1 | 0 | 0 | 0 | 1 | 1 | 1 | 1 | 1 | 0 | 1 | 0 |
| E1 | 0 | 0 | 1 | 1 | 0 | 0 | 0 | 1 | 1 | 1 | 1 | 0 | 1 | 0 |
| W5 | 1 | 1 | 0 | 0 | 0 | 0 | 0 | 1 | 1 | 0 | 0 | 0 | 0 | 1 |
| C5 | 1 | 0 | 1 | 0 | 0 | 0 | 0 | 1 | 1 | 0 | 0 | 0 | 0 | 0 |
| H5 | 1 | 0 | 1 | 0 | 0 | 0 | 0 | 1 | 0 | 0 | 0 | 0 | 0 | 0 |

**Clustering notes:** Cluster 1 (H3–E3): *S.* Kentucky ST198 + IncHI2 + *qnrS1* + *aac(6′)-Ib-cr* (global epidemic clone),Cluster 2 (H1–C1): *S.* Typhimurium ST19 + IncHI2 + *blaCTX-M-15* + IntI1 (ESBL transmission), Cluster 3 (H2–E1): *S.* Enteritidis ST11 + IncI1 + *spvB* + *pefA* (high virulence).

**Supplementary Figure S1. Full demographic data of all human participants (N = 90)**

**Supplementary Figure S2. Detailed characteristics of feed, water, and egg samples**

**Supplementary Figure S3. Detailed results of resistance genes in each *Salmonella* serovar by gel electrophoresis**
